# Ratiometric delivery of two therapeutic candidates with inherently dissimilar physicochemical property through pH-sensitive core–shell nanoparticles targeting the heterogeneous tumor cells of glioma

**DOI:** 10.1080/10717544.2018.1474974

**Published:** 2018-06-05

**Authors:** He-Lin Xu, Zi-Liang Fan, De-Li ZhuGe, Meng-Qi Tong, Bi-Xin Shen, Meng-Ting Lin, Qun-Yan Zhu, Bing-Hui Jin, Yasin Sohawon, Qing Yao, Ying-Zheng Zhao

**Affiliations:** a Department of Pharmaceutics, School of Pharmaceutical Sciences, Wenzhou Medical University, Wenzhou City, China;; b School of International Studies, Wenzhou Medical University, Wenzhou City, China;; c First Affiliated Hospital of Wenzhou Medical University, Wenzhou City, China

**Keywords:** Ratiometric delivery, dissimilar pharmacokinetics, combination therapy, heterogeneity, targeting

## Abstract

Currently, combination drug therapy is one of the most effective approaches to glioma treatment. However, due to the inherent dissimilar pharmacokinetics of individual drugs and blood brain barriers, it was difficult for the concomitant drugs to simultaneously be delivered to glioma in an optimal dose ratio manner. Herein, a cationic micellar core (Cur-M) was first prepared from d-α-tocopherol-grafted-ε-polylysine polymer to encapsulate the hydrophobic curcumin, followed by dopamine-modified-poly-γ-glutamic acid polymer further deposited on its surface as a anion shell through pH-sensitive linkage to encapsulate the hydrophilic doxorubicin (DOX) hydrochloride. By controlling the combinational Cur/DOX molar ratio at 3:1, a pH-sensitive core–shell nanoparticle (PDCP-NP) was constructed to simultaneously target the cancer stem cells (CSCs) and the differentiated tumor cells. PDCP-NP exhibited a dynamic diameter of 160.8 nm and a zeta-potential of –30.5 mV, while its core–shell structure was further confirmed by XPS and TEM. The ratiometric delivery capability of PDCP-NP was confirmed by *in vitro* and *in vivo* studies, in comparison with the cocktail Cur/DOX solution. Meanwhile, the percentage of CSCs in tumors was significantly decreased from 4.16% to 0.95% after treatment with PDCP-NP. Overall, PDCP-NP may be a promising carrier for the combination therapy with drug candidates having dissimilar physicochemical properties.

## 1. Introduction

Treatment of glioma with chemotherapy has been successful and encouraging but tumor recurrence and metastasis formation remain the major nemeses of cancer treatment. Cancer stem cells (CSCs), also called tumor-initiating cells or stem-like cancer cells are associated with tumor metastasis and recurrence after treatment. Even though many antitumor therapies eliminate the bulk of tumor cells, they may ultimately fail as they do not eradicate the CSCs which survive to regenerate new cancerous cells. CSC represents a versatile phenotypically distinct and aggressive subpopulation of tumor cells that usually display profound chemo- and radio-resistance, which contribute to a major obstacle for the treatment of cancer (Singh & Settleman, 2010). In gliomas, these cells are variously termed as they are distinguished by their characteristic markers such as the cell surface antigens CD133^high^, Nestin or ALDH1 enzymatic activity. CSCs often overexpress drug efflux transporters, spend most of their time in non-dividing G_0_ cell cycle state and can therefore escape conventional chemotherapies. The poor prognosis and outcomes of conventional therapeutic approaches provide the proof-of-concept that simultaneously targeting bulk tumor cells and a small population of CSCs may produce effective treatment of cancer and result in long-term tumor remission.

A few small molecular agents that can target CSCs has been reported in publications, including curcumin, piperine, all-trans-retinoic acid (ATRA), thioridazine, and salinomycin in various types of cancer. For instance, curcumin being a hydrophobic natural polyphenol derived from the rhizomes of turmeric (Curcuma longa) has been suggested to have a putative anti-cancer property (Bose et al., 2015). In the past few years, numerous studies have suggested that curcumin hold the potential for targeting the self-renewal pathways of CSCs in a direct or indirect way (Adiwidjaja et al., 2017). Curcumin has shown to inhibit the tumor sphere formation, serial passaging and fraction of ALDH^+^ cells in normal and malignant breast cells (Kakarala et al., 2010). Curcumin has shown to prevent NF-kappa B binding, down regulate apoptosis inhibitors and induce apoptosis as well as preventing clonogenicity in pancreatic CSCs (Kallifatidis et al., 2009; Chung & Vadgama, 2015).

Recently, the combination therapy based on usage of two or more drugs for eliminating both the bulk tumor cells and the rare CSCs has been considered to be a popular regimens for cancer chemotherapy. Ke et al. developed a mixed micelles to simultaneously encapsulate thioridazine and doxorubicin (DOX) to target both cancer cells and stem cells and thus being more effective on overcoming tumor drug resistance and relapse (Ke et al., 2014). Shen et al. (2015) adopted cationic copolymers of PCL-b-PPEEA and platinum(IV)-conjugated Pt(IV)-PCL-b-PEG to develop an approach for the effective treatment of HCC by combining platinum drugs and Notch1-targeting siRNA within one nano-assembly.

Anticancer drug combinations can act synergistically or antagonistically against tumor cells *in vitro* depending on the ratios of the individual agents comprising the combination. For example, a study has systematically examined three different drug combinations representing a range of anticancer drug classes with distinct molecular mechanisms (irinotecan/floxuridine, cytarabine/daunorubicin, and cisplatin/daunorubicin) for drug ratio-dependent synergy (Mayer et al., 2006). It was found that synergistic interactions were observed *in vitro* at certain drug/drug molar ratio ranges (1:1, 5:1, and 10:1, respectively) in each case, whereas other ratios were agonistic or antagonistic. To achieve maximal effect, it is expected that multiple drugs should be simultaneously delivered to the same cancer cell at an optimized ratio to obtain synergistic effects intracellular.

Due to the inherent dissimilar pharmacokinetics of individual drugs and the blood–brain barrier, it is difficult for the concomitant drugs to simultaneously be distributed to the glioma in an optimal dose ratio manner. Ratiometric delivery of two or more drugs by using nanoparticles has demonstrated to be an elegant and efficient approach for cancer therapy. All-trans-retinoic acid, a powerful differentiation agent of CSCs and DOX are simultaneously encapsulated in the same nanoparticle by a single emulsion method. It is demonstrated that ATRA and DOX simultaneous delivery-based therapy at a dose ratio of 3:1 can efficiently deliver the drugs to both non-CSCs and CSCs to differentiate and kill the cancer cells. A DOX/salinomycin sodium molar ratio of 1:1 had the best synergistic combination index value and both of them were co-encapsulated in nanoliposomes (SLN). The dual drugs-loaded SLN could maintain a drug ratio between 1:1 and 3:1 in 12 h *in vivo*, which showed the best tumor inhibitory rate and could also significantly decrease the percentage of CSCs *in vivo* (Gong et al., 2016).

Combination therapy of Cur with DOX at optimal dose ratio has been demonstrated to remarkably enhance anticancer efficacy and suppress the adverse effects of DOX. However, the poor pharmacokinetic properties of curcumin, in particular, a low systemic bioavailability, is still an obstacle in exploring the effects of this compound in the clinical setting. Moreover, it is difficult for individual drugs having vast differences between their physicochemical and pharmacokinetic profiles to be ratiometrically delivered into the diseased tissues. As an example, the half-life of Cur (9.7 ± 2.1 h) *in vivo* is significantly different from that of DOX (2.34 ± 1.26 h). Advancements in nanotechnology have allowed for the development of advanced drug delivery systems (Sun et al., 2011). Several nanoparticle (NP) formulations such as PLGA NPs (Misra & Sahoo, 2011), mPEG-PCL micelles (Sun et al., 2014), poly(alkyl cyanoacrylate) NPs (Duan et al., 2012), PLLA NPs (Guo et al., 2014) have been developed for the co-delivery of DOX and Cur. However, the loading cargo was nonspecifically released from most of these NPs due to the slow degradation of the polymer, which resulted in a non-ratiometric delivery (Talelli et al., 2010).

In our previous study (Xu et al., 2017), a novel amphiphilic polymer, vitamin E succinate-grafted-ε-polylysine (VES-g-ε-PLL) was synthesized and assembled into a cationic ultra-small nanoparticles which could effectively encapsulate hydrophobic curcumin. It was found that the cationic nanoparticles exhibited a strong adhesive ability against the bio-surface through electrostatic interaction and significantly promoted curcumin penetrating the BBB and glioma tissues. Given the abundant amino group of the ε-polylysine block, the surface of the VES-g-ε-PLL nanoparticles may be decorated with negatively charged polymer through an electrostatic interaction (Xu et al., 2015) to construct a functional outer shell. For example, the surface of micelle of poly(l-lysine)-block-poly(l-lactide) were easily coated using a polyanionic hyaluronic acid sodium (HA) through electrostatic interaction. Alternatively, poly-γ-glutamic acid (γ-PGA) is another anionic naturally occurring homopolyamide that consists of d- and l-glutamic acid units linked by amide bond between the α-amino and γ-carboxylic acid groups. γ-PGA is water soluble, biodegradable, edible, and nontoxic toward humans and has been broadly used as materials for drug delivery. More importantly, γ-PGA could provide the drug loading (DL) site for most of first-line chemotherapeutics such as DOX or cisplatin through electrostatic interactions or carboxyl-mental ion coordination. For instance, γ-PGA combined with DOX hydrochloride to form an insoluble DOX-NP nanocomplex. Nevertheless, the outer anionic layer by the simple physical deposition would rapidly dissipate upon exposure to blood components.

**Figure 1.**
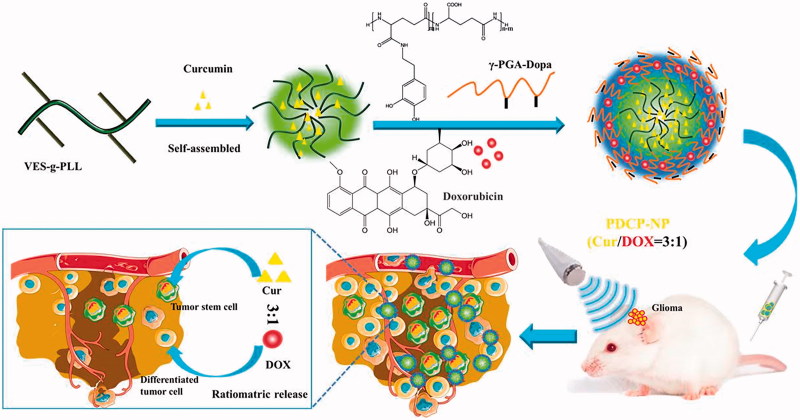
Schematic diagram of pH-sensitive core–shell nanoparticles for ratiometric delivery of curcumin and doxorubicin.

In this study, a novel dopamine-grafted-γ-PGA (γ-PGA-Dopa) was synthesized and used as the coating material. Except for the electrostatic interaction, γ-PGA-Dopa would be expected to be deposited on the surface of VES-g-ε-PLL nanoparticles through Michael addition or Schiff-mediated crosslinking reaction as mussel-inspired surface modification, constructing a pH-responsive dual-layer nanoparticle with a core–shell structure (Figure 1). To prepare the dual drug-loaded pH-sensitive micelles (PDCP-NP), the CSCs-targeting model drug, curcumin was first encapsulated into the cationic nanoparticles of VES-g-ε-PLL (Cur-NPs) and the hydrophilic DOX was encapsulated in the γ-PGA-Dopa outer shell through electrostatic interactions. Besides, γ-PGA-Dopa was expected to shield the cationic Cur-NPs core at physiological pH but shed off to re-expose the cationic Cur-NPs core for an enhanced tumor penetration. The pH-responsive dual-layer nanoparticle was carefully characterized and its ratiometric delivery ability was also carefully investigated by monitoring the ratio of Cur/Dox *in vitro* and *in vivo*. Meanwhile, *in vitro* anti-tumor effects of PDCP-NP on CSC-enriching glioma spheres were also thoroughly studied. Furthermore, in order to open the BBB, ultrasound-targeted microbubble destruction (UTMD) techniques were combined with the PDCP-NP to prove its *in vivo* antitumor effects on glioma-bearing rats. Additionally, the ability of PDCP-NP to kill the heterogeneous tumor cells in tumor tissues was also evaluated *in vitro* tumor spheroid and *in vivo* glioma-bearing rats.

## 2. Materials and methods

### 2.1. Materials

Curcumin and DOX hydrochloride were purchased from Aladdin (Shanghai, China). N-(3-dimethylaminopropyl)-N′-ethylcarbodiimide hydrochloride (EDC), N-hydroxy-succinimide (NHS), RRR-α-tocopherol succinate, dopamine, γ-polyglutamic acid (molecular weight of 90,000 Da) and fluorescein isothiocyanate (FITC) were purchased from Sigma (St. Louis, MO). Dulbecco's modified Eagle medium (high glucose) cell culture medium (DMEM) and fetal bovine serum (FBS) were purchased from Life Technologies (Grand Island, NY). C6 was obtained from the Institute of Biochemistry and Cell Biology, Shanghai Institutes for Biological Sciences, Chinese Academy of Sciences (Shanghai, China). Cell counting kit-8 (CCK-8) was purchased from Dojindo (Minato-ku, Japan). Anti-Ki67, anti-CD31, and anti-CD133 were purchased from Abcam (Abcam, Cambridge, UK). RRR-α-tocopheryl succinate-grafted-ε-polylysine conjugate (VES-g-ε-PLL) and FITC-labeled VES-g-ε-PLL were synthesized by the method depicted in our previous study (Xu et al., 2017). Dopamine-grafted-γ-polyglutamic acid was synthesized according to the procedures listed in the supplemental information (details has been depicted in Figure S1).

### 2.2. Preparation of dual-layer pH-sensitive nanoparticles (VPDP-NPs)

Dual-layer pH-sensitive nanoparticles were prepared by the following steps. First, the cationic nanoparticle core of VES-g-ε-PLL was prepared by the typical dialysis method as depicted in publication. Briefly, 500 mg VES-g-ε-PLL polymer was dissolved in 2 mL of DMSO and the polymer solution was added drop wise to 2 mL of distilled water under magnetic stirring for 10 min. The self-assembled micelle nanoparticles were dialyzed against distilled water for 48 h to remove the DMSO. The VES-g-ε-PLL nanoparticle was further lyophilized to obtain the dry powder. Second, the outer layer of γ-PGA-Dopa was deposited on the cationic nanoparticle core of VES-g-ε-PLL through pH-sensitive electrostatic coating and Michael addition or Schiff-mediated crosslinking. One hundred milligrams of VES-g-ε-PLL nanoparticles was reconstituted in 2 mL of Tris buffer (10 mM, pH 8.5) containing γ-PGA-Dopa, under continuous stirring for 3 h at room temperature (RT). Afterward, the pH of the reaction solution was re-adjusted to 7.4. In order to explore the optimal coating amount of γ-PGA-Dopa on VES-g-ε-PLL nanoparticles, a series of dual-layer nanoparticles were prepared by changing the amount of γ-PGA-Dopa in formulation.

### 2.3. Characterization of dual-layer pH-sensitive nanoparticles

Particle size, particles size distribution, and zeta potential of polymeric nanoparticles were measured by dynamic laser light scattering (DLS) using a Zetasizer Nano ZS at a laser beam wavelength of 632.8 nm and a scattering angle of 90°. The morphology of the nanoparticles was examined using an H-7500 transmission electron microscope with phosphotungstate acid solution (2%, w/w) staining. X-ray photoelectron spectroscopy (XPS, ESCALAB 250Xi) was used to identify the coating materials.

### 2.4. Co-encapsulation of curcumin and doxorubicin in pH-sensitive nanoparticles (PDCP-NP)

Two model drugs were encapsulated step by step in the dual-layer pH-sensitive nanoparticles. Briefly, the hydrophobic model drug, curcumin was loaded in VES-g-ε-PLL nanoparticles by the same procedures as described in section 2.2 by just dissolving curcumin and the polymer in DMSO. The unloaded curcumin was then removed by ultracentrifugation at a speed of 10,000 rpm to obtain the Cur-NPs solution. Similarly, the outer layer of γ-PGA-Dopa was deposited on the cationic nanoparticle core of VES-g-ε-PLL by the same procedure as the blank dual layer nanoparticles. The hydrophilic model drug, DOX hydrochloride (1 mg/mL) was then added to the γ-PGA-Dopa-coating Cur-NPs solution at a feeding ratio of *R* = 0.5 (*R* is the molar ratio of DOX/carboxy groups of γ-PGA-Dopa) and further incubated for 24 h at RT. Finally, the unencapsulated DOX in the dual drugs loaded nanoparticles was removed by dialysis against water for 12 h. As a control, two types of single drug-loaded nanoparticles including Cur-NPs and DOX-NPs were also prepared by the similar method but with the addition of only one drug.

Drug loading capability and encapsulating efficiency of the two drugs in the dual-layer nanoparticles were determined by Waters 2695 HPLC equipped with a reverse phase C_18_ column (250 × 4.6 mm, 5 μm). HPLC conditions were used as follows for analysis: acetonitrile–0.2% acetic acid (65:35, v/v) was the mobile phase for Cur analysis while acetonitrile–0.05% acetic acid (26:74, v/v) was that for DOX analysis. The flow rate was 1 mL/min and the column temperature was kept at 30 °C. Drug loading efficiency (DL %) and drug encapsulation efficiency (EE %) of Cur and DOX in micelles were calculated using the following equation:

$$DL=\frac{W_{\text{Cur/DOX}}}{W_{\text{Cur/DOX}}+W_{\text{polymer}}}$$

$$EE=\frac{W_{\text{Cur/DOX}}}{{W\prime }_{\text{Cur/DOX}}}$$

where *W*_Cur/DOX_ is the amount of curcumin or DOX assayed in micelles, *W*_polymer_ is the amount of polymer used in formulation, and *W*′_Cur/DOX_ is the amount of curcumin or DOX added in formulation.

### 2.5. *In vitro* drug release

*In vitro* release profiles of Cur or DOX from PDCP-NP were carried out in PBS containing 0.5% (w/w) Tween 80 at different pH values (7.4 and 5.0) by the dialysis method. 1.0 mL of PDCP-NP was placed into the dialysis bag (MWCO: 3.5 kDa) and was dialyzed against 10 mL of release medium under gentle stirring at 37 °C. At predetermined time intervals, 10 mL of the released medium was completely withdrawn and equivalent fresh release medium was replenished. The concentration of Cur or DOX in the release medium was determined by the HPLC assay as depicted in section 2.4 at 431 nm and 495 nm, respectively. Each sample in the release kinetics study was conducted in triplicate. The cumulative release percentage was calculated by following formula.

$$Drug\;release\;percentage\;\left(\%\right)=\frac{\sum_{0}^{t}Q_{t}}{w_{total}}\times 100$$

where *Q_t_* is the amount of curcumin or DOX released at *t* time point and *w_total_*is the total amount of Curcumin or DOX in lyophilized powder.

### 2.6. *In vitro* cells experiments

#### 2.6.1. Enrichment of cancer stem cells in glioma spheres

As previously reported tumor cell spheroids were commonly used to enrich CSCs. In order to culture C6 cell spheroids, a 48-well culture plate was pre-coated with 2% of agarose in free DMEM. A total of 2 × 10^3^ cells in 400 mL culture medium were seeded into each well. The plates were gently agitated for 5 min and cultured at 37 °C in the presence of 5% CO_2_ for several days.

To identify the cell-surface phenotype of CSCs in the spheroids, the resulting glioma spheroids were washed with PBS, embedded in Cryomatrix^TM^ and then cut into frozen sections with 8 μm-thicknesses for immunostaining. Frozen sections were first fixed with acetone at RT for 15 min, rehydrated in PBS and incubated with mouse anti-CD133 (Abcam, 1:200, Cambridge, UK) in PBS for 1 h at RT. Subsequently, the sections were washed with PBS and incubated with the secondary antibody, anti-rabbit IgG Alexa FluorVR (488) (ab150083, 1:1500, Abcam, Cambridge, UK) in PBS for 1 h. Cellular nuclei were stained by DAPI (Beyotime, Nantong, China). Meanwhile, some of the glioma spheroids were treated with trypsin (0.05% with 0.02% EDTA, Gibco, Montreal, Canada), washed twice with FACS buffer (2% FBS in D-PBS), and then incubated with the primary antibody (1:50) for 1 h on ice, followed by incubation with FITC labeled secondary antibody (1:1000) for 30 min. The cells were analyzed on FACS into a flow cytometer (Beckton Dickinson, Franklin Lakes, NJ), and the data obtained were processed using the FlowJo package (Tree Star, Ashland, OR). The digested cells treated with only the secondary antibody were used as a background control.

#### 2.6.2. *In vitro* cytotoxicity of PDCP-NP

*In vitro* cytotoxicity of PDCP-NP against C6 cells was tested by CCK8 kits. Briefly, C6 cells were cultured in DMEM medium supplemented with 10% FBS under a humidified atmosphere consisting of 5% CO_2_ at 37 °C. Afterward, the cells were treated with different formulations for 48 h or 72 h and 10 µL of CCK-8 were added into each well. After 2 h of incubation, absorptions at 450 nm wavelength were measured using a microplate reader (MultiskanMK3, Thermo, ‎Waltham, MA).

#### 2.6.3. *In vitro* cellular uptake of PDCP-NP

C6 cells in complete DMEM culture medium were seeded at a density of 1 × 10^6^ cells per well on six-well culture plates. After 24 h, they were treated with Cur/DOX DMSO solution or PDCP-NP at equivalent doses of Cur (2 μg/mL) and DOX (0.67 μg/mL). After incubation for 1 h, 4 h, or 8 h, C6 cells were rinsed with cold PBS, fixed with 4% paraformaldehyde and qualitatively analyzed using confocal laser microscopy (A1 PLUS, Tokyo, Japan).

To determine the intracellular drug content, C6 spheroid-derived cells or adhesive cells were treated with trypsin (0.05%, with 0.02% ethylene diamine tetraacetic acid, Gibco, Montreal, Canada), then washed with PBS twice and finally redispersed in 500 μL PBS. The cells were lysed with ultrasonic probe and DMSO (2 mL) was added to the lysate and centrifuged at 120,000 rpm to extract the supernatant drug. The drug contents were further determined by HPLC.

#### 2.6.4. *In vitro* growth inhibition of PDCP-NP against glioma spheroids

Uniform and compact glioma spheroids were selected for subsequent experiments. When the volume of glioma spheroids reached about 500 mm^3^, C6 glioma spheroids were treated with free Cur/DOX solution, DOX-VPDP, Cur-VPDP, and PDCP-NP, and having a final curcumin concentration of 2 μg/mL in each well. Glioma spheroids incubated in DMEM without any treatment served as control. After these treatments, glioma spheroids were observed with an inverted microscope (Chongqing Optical & Electrical Instrument Co. Ltd., Chongqing, China). The maximum diameter (*d*_max_) and minimum diameter (*d*_min_) of each spheroid were measured every day for three days. The spheroid volume was calculated using the following formula: $V=0.5\times d_{max}\times {d_{min}}^{2}$

#### 2.6.5. *In vitro* imbibition of CSCs inside glioma spheroids after treatment with PDCP-NP

Spheroids treated with different formulations for three days were washed with PBS and subsequently embedded in Cryomatrix^TM^. The resulting spheroid array was cut into 8 μm-thick sections and processed for immunostaining. Cryosections were first fixed with acetone at RT for 15 min, rehydrated in PBS and incubated with mouse anti-CD133 (Abcam, 1:200, Cambridge, UK) in PBS for 1 h at RT. Subsequently, the sections were washed again with PBS and incubated with secondary antibody anti-rabbit IgG Alexa FluorVR(488) (ab150083, 1:1500, Abcam, Cambridge, UK) for 1 h. The nuclei were stained using DAPI (Beyotime, Nantong, China).

For flow cytometry analysis, C6 glioma spheroids cells were washed with FACS buffer (2% FBS in D-PBS) at a rate of 1 million cells per mL. The cells were incubated with primary antibody (1:50) for 1 h on ice, washed thrice with FACS buffer, incubated with FITC conjugated secondary antibody (1:1000) for 30 min on ice and washed thrice again with FACS buffer. Cells incubated with only the secondary antibody were used as a background control. The cells were analyzed on FACS into a flow cytometer (Beckton Dickinson, Franklin Lakes, NJ) and the obtained data processed using the FlowJo package (Tree Star, Ashland, OR).

Uniform and compact glioma spheroids were selected for subsequent experiments. Primary spheroids were treated on day 6 after seeding of the wells (the time at which well-formed primary-spheroids were present). Free Cur/DOX solution (3:1), DOX-VPDP, Cur-VPDP, and PDCP-NP (3:1) were added into each well and incubated for three days with an equivalent concentration of Cur (9 μg/mL). For generating secondary spheroids, wells containing primary spheroids were enzymatically dissociated and approximately 5 × 10^3^ cells were re-plated in low-attachment plates.

#### 2.6.6. Penetration of PDCP-NP against glioma spheres

To evaluate the penetration ability of PDCP-NP into the glioma spheroids, the glioma spheroids were treated with Cur/DOX solution, DOX-VPDP, Cur-VPDP, and PDCP-NP for 24 h, respectively. The final curcumin concentration was 2 μg/mL in each well. The glioma spheroids were rinsed three times with PBS, fixed with 4% paraformaldehyde, transferred to a chambered covered slip and observed by confocal microscopy (A1 PLUS, Japan).

### 2.7. *In vivo* anti-tumor effect of PDCP-NP

#### 2.7.1. Establishment of rat model bearing in situ glioma

All animal experiments were performed under the approval and guidance of the Institutional Animal Care and Use Committee of Wenzhou Medical University. Male Sprague-Dawley rats (250–350 g) were purchased from Shanghai Lab. Animal research Center (Shanghai, China). The *in situ* glioma models were established according to the reported method. In brief, rats were deeply anesthetized with 10% chloralic hydras (i.p. 400 mg/kg) and immobilized on a stereotactic frame (KOPF900, Tijunga, CA). A sagittal incision was made through the skin overlying the calvarium, and a small dental drill was used to create a hole (1.0 mm diameter) in the cranium 1.0 mm posterior to the bregma and 3 mm lateral to the sagittal suture (right hemisphere of the brain) without duramatral damage. Subsequently, approximately 1 × 10^6^ cells/10 μL of C6 glioma cell suspension was injected into the hole. The needle fell 6.0 mm followed by a 1.0 mm rise perpendicular to the hole such that the net depth of the injection needle in the hole was 5.0 mm from the brain surface. The injection was performed over a 10-min period. The growth of rat brain tumors was monitored longitudinally using MRI.

#### 2.7.2. *In vivo* anti-GBM effect

To evaluate for the anti-tumor effects of PDCP-Micelles *in vivo*, glioma rats were randomly divided into five groups (eight rats per group). Treatment began on the 7th day after tumor implantation and all treatments were intravenously administered. Glioma rats in the control group were administrated with saline and the other five groups were administrated with Cur/DOX solution, DOX-loaded nanoparticle (DOX-VPDP), curcumin-loaded nanoparticle (Cur-VPDP), and PDCP–NP. All formulations blending with microbubble (MB) were administered prior to US treatment. The dose of curcumin and DOX in the combination therapy groups was 1 mg/kg and 0.33 mg/kg, respectively. The concentration of phospholipid-based microbubbles in the final injection was 1 × 10^6^ microbubbles/mL. All glioma rats were anesthetized with chloral hydrate (350 mg/kg body weight) 5 min before the experiment. Ultrasound transmission gel was applied on the surface of the rat brain corresponding to the glioma. Experimental solution was infused via the tail vein through a 20-gauge cannula. Immediately after the intravenous injection, a linear array transducer (Acuson Sequoia 512C system, Siemens, Munich, Germany) was applied to generate the UTMD effect (MI = 1.9, exposure time = 10 s).

Tumor progression was assessed by MRI with a 3T scanner once every week and the tumor volume was also calculated by MRI. Tumor sizes were evaluated with image analysis software using MATLAB (Math-Works, Natick, MA). Tumor volumes (*V*) were calculated using an ellipsoid approximation (*V* ≈ 4/3 × π × (0.5)^3^× (*abc*)], where *a*, *b*, and *c* are the maximum diameters of the tumor measured in three orthogonal planes on 2-D T2-weighted MR images. The mean survival times of rats from each group were monitored and analyzed.

#### 2.7.3. *In vivo* bio-distribution

Ten days after the implantation of C6 cells, the glioma-bearing rats were intravenously administrated with PDCP-NP (1 mg/kg of equivalent Cur, 0.33 mg/kg of DOX) through their tail veins. The rats were executed 2 h after injection and the major organs including the heart, liver, spleen, lungs, kidneys, and brain were collected for *ex vivo* imaging (CRi maestro, Woburn, MA). These tissues were then frozen and sliced into thin section having thicknesses of 5 μm in order to observe the fluorescent drugs by confocal laser microscopy (A1 PLUS, Japan). Meanwhile, these tissues were also weighed and homogenized for quantitative determination of the amount of Cur and DOX by HPLC as previously mentioned.

### 2.8. Histopathological evaluation of glioma tissue after treatment with PDCP-NP

#### 2.8.1. Histological morphology of glioma

After completion of the treatments, the rats were sacrificed and their brains and other organs were collected. The brain tumors were fixed in 10% buffered formalin, embedded in paraffin and then sectioned at 5 mm thicknesses. The sections were stained with H&E. The tumor histology was viewed and imaged under optical microscopy (Nikon ECLIPSETi-S, Ruikezhongyi Company, Beijing, China).

#### 2.8.2. Tunnel staining of glioma tissues

Apoptotic cells in glioma were detected via TUNEL assays using an *in situ* cell death detection kit, following the manufacturer's instructions prior to observation under light microscopy.

#### 2.8.3. Immunohistochemical staining

To further explore the therapeutic mechanism of the PDCP-NP, immunohistochemical staining of glioma was performed for the detection of Ki67 or CD31 after the different treatments. The antibodies used were rabbit monoclonal antibody to Ki67 (1:200, Abcam, Cambridge, UK) or CD31 (1:200, Abcam, Cambridge, UK). Paraffin-embedded tumors were cut into 5-mm-thick sections, de-paraffinized in xylene series and hydrated in distilled water. Antigen retrieval was obtained by the citrate buffer and washed with PBS. Samples were blocked with 3% H_2_O_2_ for 5 min, followed by treatment with primary antibodies for 2 h at 37 °C, HRP-secondary antibody for 30 min at 37 °C and were then stained with DAB and counterstained with hematoxylin. The stained sections of the brain were examined and recorded with an optical microscope. The number of stained cells was evaluated by manual counting of four random microscopic fields (400× original magnification) per subject.

Immunofluorescence staining of the CSC markers (CD133) were also performed on deparaffinized sections. Sections were stained with anti CD133 followed by goat anti-rabbit IgG Alexa FluorVR(488) (ab150083, 1:1500, Abcam, Cambridge, UK). The nuclei were stained using DAPI (Beyotime, Nantong, China). Angiogenesis was observed by confocal laser microscopy (A1 PLUS, Japan).

#### 2.8.4. Population of CSCs in glioma after treatment with PDCP-NP

At the conclusion of the tumor suppression study, the animals were sacrificed and tumor tissues were excised. The tumor tissues were transferred to a dish and cut into small pieces. The fragments were suspended in 20 mL of DMEM medium and collected by centrifugation at 600 rpm for 5 min. The pelleted materials were suspended in 10 μL of tumor cell digestion solution (1 mg/mL collagenase Type I in PBS, Invitrogen, Carlsbad, CA) and incubated at 37 °C for 3 h with persistent agitation. The tumor cells were collected by centrifuging at 1200 rpm for 6 min at RT and then washed twice with PBS containing 1% FBS. The tumor cells were filtered twice through a 200-mesh sieve, stained with CD133 substrate as described above for FACS analyses.

### 2.9. Systemic toxicity of PDCP-NP against healthy rats

To evaluate the toxicity of the PDCP-NP, samples of heart, liver, spleen, lung, and kidney tissue were collected and routinely stained with hematoxylin and eosin (HE) after the rats were sacrificed.

### 2.10. Statistical analysis

The sizes of tumors were evaluated with image analysis software using MATLAB (Math-Works, Natick, MA). Least-squares nonlinear regression analyses were performed to compare the rates of tumor growth between the groups. Population survival curves were also plotted using the Kaplan–Meier method. Survival curves were compared between groups using the log-rank test. Statistical analyses were performed using GraphPad Prism version 5.01 for Windows (GraphPad Software, San Diego, CA). All results are expressed as mean ± SD for each group. Student's *t*-test was performed to determine significant differences between two groups. One-way analysis of variance (ANOVA) was used to determine the significant differences among multiple groups and *p* < .05 was considered statistically significant.

## 3. Results

### 3.1. Preparation and characterization of pH-sensitive nanoparticles with dual-layer structure

The pH-sensitive nanoparticles with dual-layer structure (VPDP) were prepared by formulating a cationic VES-g-ε-PLL nanoparticle core (VP) followed by deposition of the negative γ-PGA-Dopa outer layer through pH-sensitive electrostatic coating and Michael addition or Schiff-mediated crosslinking. As shown in Figure S2(A) (supplementary information), a single peak with a mean *D*_h_ of 19.3 nm for the VES-g-ε-PLL nanoparticle was observed by DLS analysis. However, after coating with γ-PGA-Dopa, two peaks were observed in most of the formulations. A new peak at ca. 100 nm was attributed to the coated nanoparticle (VPDP) while the primary peak at ca. 20 nm for the VP nanoparticle, indicating the incomplete coating of VP nanoparticles in these formulations. But as the feed ratio of γ-PGA-Dopa/VP increased, the peak of the VP nanoparticle decreased and the peak of VPDP became more obvious. At a feed ratio of 1:2, the particle size distribution became a single peak distribution and the primary VP peak disappeared, suggesting the complete coating of the VP nanoparticles. Alternatively, zeta potential of nanoparticles after γ-PGA-Dopa coating also showed a dramatic conversion in function of the γ-PGA-Dopa/VP feeding ratio (Figure S2(B)). The zeta potential of the VP nanoparticle was positive at 17.3 ± 2.87 mV, while a transition of the zeta potential from positive to negative was observed after γ-PGA-Dopa coating and the zeta potential of VPDP decreased with increasing γ-PGA-Dopa/VP feeding ratio. As the γ-PGA-Dopa/VP feeding ratio was approaching 1:2, the lowest zeta potential of VPDP was at –37.6 ± 6.12 mV, further confirming the complete coating of the VP nanoparticle with γ-PGA-Dopa. The morphology of VPDP at γ-PGA-Dopa/VP feeding ratio of 1:2 was also observed by TEM. As shown in Figure S2(C), VP nanoparticles exhibited a uniform spherical morphology with diameters of ca. 15 nm, which was also close to the hydrodynamic diameter (19.3 nm) determined by DLS. By contrast, the TEM graph of the VPDP nanoparticles exhibited a sphere-like dual-layer structure with a gray coating layer around a dense core (Figure S2(D)). The dry particle size determined by TEM was ca. 100 nm, which was smaller than that determined by DLS (ca. 122 nm). This was because of the dehydration of the hybrid-core under a high vacuum before TEM was measured (Xu et al., 2015). In view of the uniform particle size distribution and complete packaging of VES-g-ε-PLL micelles, VPDP nanoparticle at VP/γ-PGA-Dopa of 2:1 was used for further encapsulation of the model drugs.

### 3.2. X-ray photoelectron spectroscopy of VPDP nanoparticles

**Figure 2.**
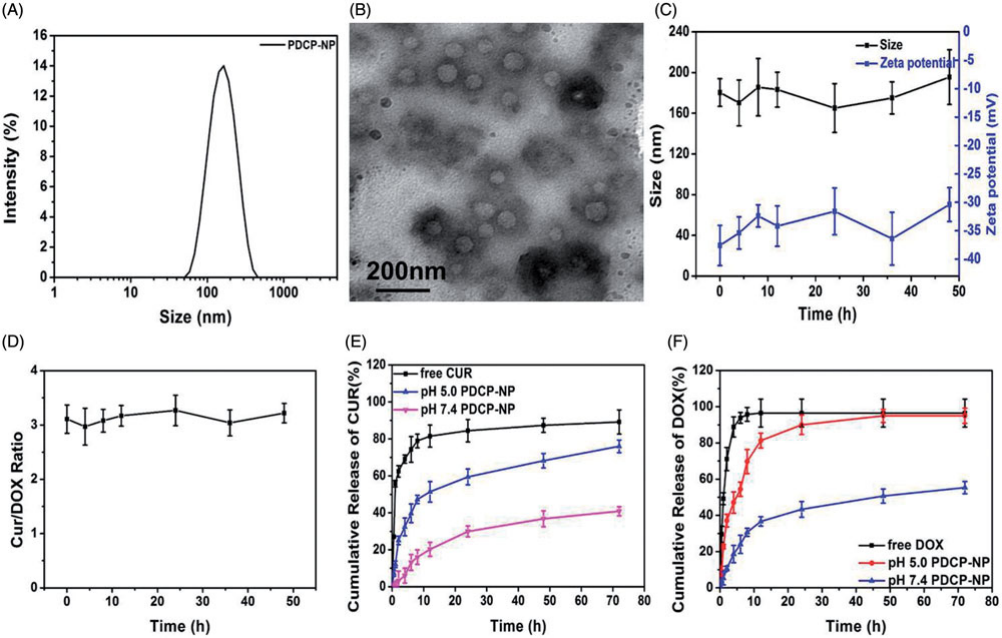
(A) DLS and (B) TEM graphics of PDCP-NP in pH7.4 PBS of PDCP-NP, (C) the size/Zeta potential of PDCP and (D) the dose ratio of Cur/DOX in PDCP at different time interval during incubating with physiology-mimicking medium containing 10% FBS at 37 °C, and the cumulative release profiles of Cur (E) and DOX (F) from PDCP in pH7.4 PBS or pH 5.0 acetate buffer. Results are expressed as the mean ± SEM (*n* = 3).

To confirm the cross-linking reaction within the outer coating, the XPS spectrum of the VPDP nanoparticle was analyzed and the results are shown in Figure 2. Although both of the VP nanoparticle and the VPDP nanoparticle exhibited a group of peaks associated with C1s, O1s, and N1s elements, the XPS spectrum of VPDP was closer to that of the γ-PGA-Dopa polymer (Figure 2(A)). Besides, the O/N ratio in the VPDP nanoparticle was significantly different from that of the VP nanoparticle. The O/N ratios are shown in Figure 2(B). The O/N ratio of the VP nanoparticle was calculated to be 1.16 according to the XPS results, indicating an enrichment of amino groups on its surface. The O/N ratio of the simple physical mixture between the VP nanoparticle and the γ-PGA-Dopa polymer was 1.24, which was not significantly different from that of the VP nanoparticle. However, after coating with γ-PGA-Dopa polymer, the O/N ratio of VPDP nanoparticles increased to 1.75, indicating an effective shielding of the surface of the VP nanoparticles provided by γ-PGA-Dopa. The increment in O/N ratio of VPDP could be due to the electrostatic interaction between the carboxyl groups of γ-PGA-Dopa and the amino groups presented on the surface of the VP nanoparticles.

Apart from this, VPDP nanoparticles exhibited different C1s peaks from VP nanoparticles (Figure 2(C)). The C1s peaks of VP nanoparticles exhibited three peaks at around 284.2 eV, 285.1 eV, and 287.8 eV, which were assigned to the C–C, C–N, and C-O components, respectively (Hunke et al., 2015). The C1s peaks of the γ-PGA-Dopa polymer were composed of four peaks at around 284.0, 284.9, 286.8, and 287.8 eV, which can be generally assigned to the C–C, C–OH, C–O, and C-O components, respectively (Liu et al., 2015). A simple overlap for these peaks was observed in the spectrum of the physical mixture between the VP nanoparticle and γ-PGA-Dopa polymer. However, the peak attributed to the C–N component disappeared and the peaks attributed to C–C component became intense in the VPDP nanoparticles, indicating the crosslinking reaction within the coating layer. It was reported that dopamine residues in γ-PGA-Dopa oxidize to quinone in a basic medium, which can further react with the free amino groups on surface of the VP nanoparticles or form an aryloxy radical to induce subsequent polymerization between catechol groups (Ochs et al., 2011) (Figure 2(D)).

### 3.3. Simultaneous encapsulation of curcumin and doxorubicin in VPDP nanoparticles

The hydrophobic curcumin was encapsulated into the hydrophobic core of VP micelles to obtain curcumin-loaded VP (Cur-VP). Afterwards, the negative γ-PGA-Dopa layer was deposited on the surface of Cur-VP in a basic medium to prepare Cur-VPDP by the same methods used to prepare the blank VPDP nanoparticles. Doxorubicin hydrochloride was encapsulated into the shell of Cur-VPDP at a predetermined dosage ratio of Cur/DOX through electrostatic interaction to formulate the dual drugs loaded nanoparticle (PDCP-NP). The basic properties of PDCP-NP were also carefully characterized and results are shown in Supplementary Table S1. After the simultaneous encapsulation of Cur and DOX, *D*_h_ of PDCP-NP slightly increased to 160.8 nm in comparison with the blank VPDP (*D*_h_ of 122.4 nm), while the zeta potential increased from –37.6 mV (VPDP) to –30.5 mV (PDCP-NP) because of the neutralization of DOX on the carboxyl groups of the γ-PGA layer on the surface of VP (Cho et al., 2016). Clearly, the most suitable particle sizes suitable for accumulation in tumors correspond to that of PDCP-NP (100–200 nm) as an enhanced permeability and retention effect (EPR) is provided and a negative zeta potential would be helpful for a prolonged circulation time in the bloodstream. Moreover, the particle size distribution of PDCP-NP became narrower than that of VPDP as shown in Figure 2(A). A spherical morphology with a core–shell structure was also observed in the TEM graphic of PDCP-NP (Figure 2(B)) but its coating shell after the encapsulation of DOX was denser than that of VPDP. The DL of Cur and DOX in PDCP-NP was determined to be 4.46 ± 0.87% (Cur) and 1.47 ± 0.49% (DOX), respectively. The dose ratio of Cur/DOX in PDCP-NP was close to 3:1(w/w) and the entrapment efficiency (EE) for each model drug was more than 85%. The stability of PDCP-NP in a physiology-mimicking medium was also evaluated by detecting its particle size, zeta potential and leakage of the loading drug. As shown in Figure 2(C), the particle sizes remained in the range of 140–160 nm during the whole 48 h of incubation and there was also no significant variation in their zeta potentials, which may indicate a good stability of PDCP-NP in blood. Moreover, the dose ratio of Cur/DOX in PDCP-NP throughout the incubation did not change from the initial ratio of 3:1 (Figure 2(D)), further indicating a strong stability of the payload.

### 3.4. *In vitro* drug release

*In vitro* release profiles of Cur and DOX from PDCP-NP were investigated using a dialysis method at 37 °C in pH 7.4 PBS or pH 5.0 acetate buffer containing 0.5% (v/v) Tween-80. The results were shown in Figure 2(E,F). The sustained-release profile of the loaded Cur or DOX from PDCP-NP was observed in pH 7.4 PBS. Even at 72 h, the cumulative drug release of Cur and DOX from PDCP-NP was only 40.92 ± 2.45% and 80.27 ± 3.43% (DOX), respectively. However, a pH-sensitive release behavior for both Cur and DOX from PDCP-NP was exhibited when exposed to the release medium having a pH of 5.0. More than 75% of the loaded Cur and 95% of the loaded DOX were released from PDCP-NP in pH 5.0 release medium at 72 h. The pH-sensitive release profiles may be associated to the following reasons. First, the side carboxyl groups of the coating γ-PGA layer on PDCP-NP would be immediately protonated when exposed to the acidic condition, which would then result in a loss of electrostatic interactions between the coating layer and DOX. This would cause a rapid release of DOX from the nanoparticles. Alternatively, the chemical crosslink between the free amino group and the dopamine residues of γ-PGA-Dopa was easily cleaved in the pH 5.0 medium, which caused the coating layer to detach and subsequently expose the fine Cur-VP micelles. Extensive protonation of the free amino group on the exposed Cur-VP micelles in the acidic medium would accelerate the release of Cur from PDCP. Furthermore, the morphology of PDCP-NP during the release was monitored by TEM and the results are shown in Figure S2. The spherical nanoparticles were still observable in the pH 7.4 release medium (Figure S3(A)), which indicated that the morphological structure of PDCP-NP was still kept intact in this release medium. However, the coating layer on PDCP-NP was destroyed and a flocculate precipitate was formed in pH 5.0 release medium (Figure S3(B)). Interestingly, some of fine VP nanoparticles were truly observed and buried in the precipitate. The protonation of the side carboxyl groups on the γ-PGA block and the destruction of the crosslink between the VP nanoparticle and the γ-PGA-Dopa in an acidic environment were also reported in these publications (Mayer et al., 1990; Xu et al., 2015).

### 3.5. *In vitro* cytotoxicity of PDCP against glioma cells

#### 3.5.1. Enrichment and identification of heterogeneous cells in glioma spheroid

3D tumor cells spheroid-forming culture is a common method to enrich CSCs. In this study, C6 spheroid was successfully cultured on 2% agarose hydrogel to enrich BTSCs. When the size of the cell spheroids grew up to approximately 500–600 μm after seven days, BTSCs in the C6 spheroids were sorted and identified by the surface markers (CD133^+^). As shown in Figure S5(A), CD133^+^ cells became more obvious in 3D glioma spheroids, while there was a scarcity of BTSC in the 2D adherent cells. BTSCs in 3D cell spheroids were further quantitatively determined by flow cytometry and the percentage of CD133^+^ cells was significantly increased from 0.48% for the 2D adherent cells culture to 17.85% for the 3D cell spheroids (Figure S5(B)).

#### 3.5.2. Dosage ratio-dependent cytotoxicity of cur/DOX on glioma spheroid

It was reported that spheroid-derived cells were more resistant to the common chemotherapeutics than the popular culture-derived cells (Sun et al., 2015). The cytotoxicity against spheroid-derived C6 cells was evaluated and the results are displayed in Figure S6. Glioma spheroid-derived C6 cells were less sensitive to the combinational Cur/DOX solution than the common C6 cells, indicating a strong drug-resistant of the spheroid-derived C6 cells. However, when the dose ratio of Cur/DOX in the formulation increased from 1:3 to 3:1, its cytotoxicity against spheroid-derived C6 cells became more obvious. The strongest cytotoxicity against tumor spheroid-derived C6 cells was observed when the dose ratio of Cur/DOX was at 3:1.

**Figure 3.**
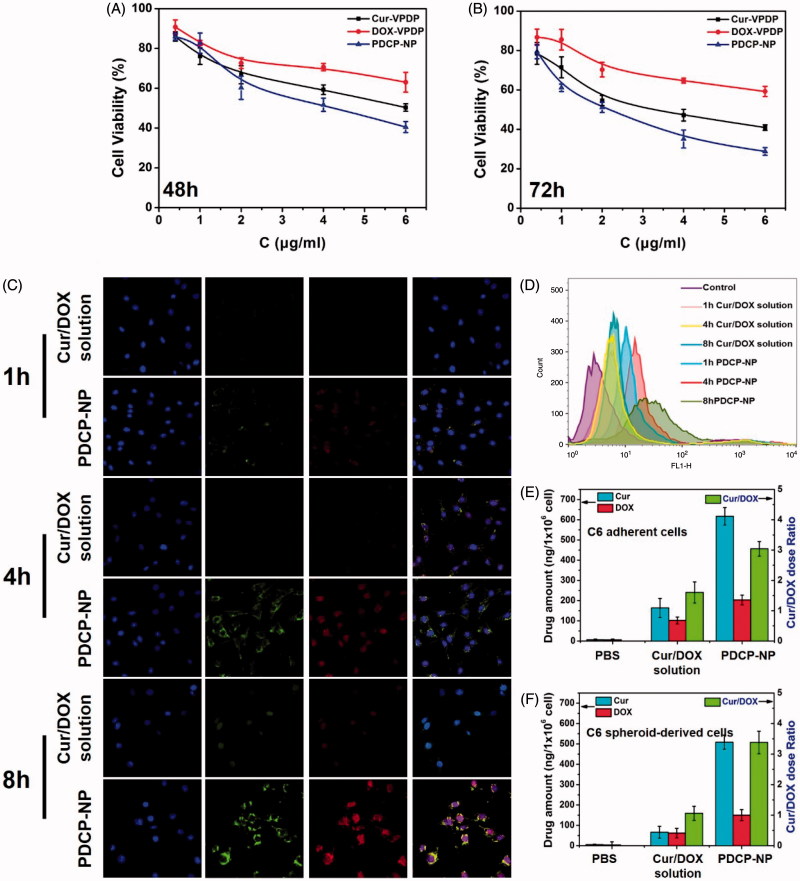
*In vitro* cytotoxicity of Cur-VPDP, DOX-VPDP, or PDCP-NP against glioma spheroids-derived C6 cells at total drug concentrations of 0.4–6 μg/mL after 48 h (A) and 72 h (B), (C) CLSM images and flow cytometry (D) of glioma spheroids-derived C6 at 1 h, 4 h, and 8 h after treatment with the combinational Cur/DOX solution and PDCP-NP at Cur concentration of 1 μg/mL and DOX of 0.33 μg/mL, and the intracellular Cur and DOX concentrations of adherent (E) and spheroid-derived (F) cells were determined by HPLC after 8 h of incubation with various formulations. Data are shown as mean ± SD (*n* = 3), original magnification: 600×.

To improve the anti-tumor effect of the combined Cur/DOX, both Cur and DOX were simultaneously encapsulated into our designed VPDP nanoparticles at a precise Cur/DOX dose ratio of 3:1 to obtain the optimal PDCP-NP. The cytotoxicity of the PDCP-NP against spheroid-derived C6 cells was also evaluated *in vitro* using the CCk-8 kit. As shown in Figure 3(A,B), PDCP-NP demonstrated a time and dose-dependent characteristic in inhibiting the proliferation of spheroid-derived C6 cells. Moreover, PDCP-NP exhibited a stronger cytotoxicity than signal drug-loaded nanoparticles (Cur-VPDP or DOX-VPDP) or the combinational Cur/DOX solution.

#### 3.5.3. Ratiometric cellular uptake of the combined cur and DOX in PDCP-NP by glioma spheroid

*In vitro* cellular uptakes of PDCP-NP by spheroid-derived C6 cells were further investigated and the results are shown in Figure 3(C). As expected, there was an easy uptake of PDCP-NP by the spheroid-derived C6 cells in a time-dependent manner but little of the combinational solution of Cur/DOX entered the intracellular compartment. After incubation, both the green fluorescence emitted by Cur and red fluorescence emitted by DOX were uniformly distributed in the cytoplasm around the cellular nucleus for the PDCP-treated spheroid-derived C6 cells and their fluorescent intensities increased with incubation time. Interestingly, when incubation time increased to 8 h, most of the Cur and DOX permeated into the cellular nucleus where a strong fluorescence was distributed. By contrast, after incubation with the combined Cur/DOX solution, a faint red fluorescence was observed only inside the cellular nucleus at the 4 h mark. Furthermore, the fluorescence was not significantly intensified in this compartment even after 8 h of incubation. The cellular uptake of PDCP-NP was further quantified by flow cytometry and the results shown in Figure 3(D) exhibited similar results. The total drug amount internalized by spheroid-derived C6 cells in PDCP-NP was significantly higher than that in the Cur/DOX solution, indicating that the nano-scale drug vehicles facilitated the uptake of both of DOX and Cur.

In order to confirm whether Cur/DOX was ratiometrically internalized by the spheroid-derived C6 cells, the intracellular amount of Cur and DOX after 8 h of incubation with PDCP-NP was quantitatively analyzed by HPLC and the dose ratio of Cur/DOX was calculated accordingly. As shown in Figure 3(E–F), the intracellular Cur/DOX dose ratio was calculated to be 3.3:1, which was close to the theoretical ratio of Cur/DOX (3:1). By contrast, either the internalized amount of Cur or the amount of DOX was very low. The intracellular ratio of Cur/DOX in the cytoplasm was calculated to be 1:1, which was significantly different from the theoretical ratio. The ratiometric cellular uptake of both drugs in the form of PDCP might indicate that the intact nanoparticles were secured in the cytoplasm of C6 cells instead of being prematurely released.

The intracellular fate of PDCP-NP post cellular uptake was also tracked by TEM techniques at different time points. As shown in Figure S6, most of the PDCP-NP (red arrow) was localized inside cellular membrane after 10 min of incubation. As the incubation time increased to 1 h, some fine nanoparticles together with flocculate (yellow arrow) were observed in the cytoplasm, which may reflect the detachment of the pH-sensitive coating layer from the surface of PDCP to exposure its Cur-VP core in the acidic endosome. Interestingly, after 8 h of incubation, most of fine nanoparticles were localized inside the nucleus and some nanoparticles were distributed along the nucleolus membrane. These observations may suggest that the exposed Cur-VP was able to enter the cellular nucleus via the nucleopore. The intra-nuclear transporting ability of Cur-VP may be associated to the micelles-forming composite (VES-g-ε-PLL) having a high affinity to the intra-nuclear DNA. PLL has been reported to act as a molecule with nuclear localization signal for intra-nuclear transport in literature (Fan et al., 2016).

### 3.6. Inhibitory effect of PDCP against glioma spheroid *in vitro*

#### 3.6.1. Inhibitory effect of PDCP on growth of glioma spheroid *in vitro*

When cells spheroid gradually grow to approximately 500 μm of diameter, the inhibitory effect of PDCP-NP on growth of glioma spheroid was also evaluated. Microscopic images of the glioma spheroids were monitored and their volumes were calculated after treatment. The results are exhibited in Figure S8(A&C). Just on the third day after treatments, it was observed that the tumor spheroids with PBS grew rapidly, with volumes as high as 1.75 times its original volume. A slight growth delay of tumor spheroids was observed in the group treated with free Cur/DOX solution or DOX-VPDP. The volumes of spheroids on day 3 were 1.48- and 1.34-times that of the original volume after treatment with Cur/DOX solution and DOX-VPDP, respectively. In contrast, a sharp growth delay was observed after treatment with Cur-VPDP or PDCP-NP. The volumes of glioma spheroids on day 3 decreased by 0.85 and 0.63 folds of its initial size after treatment with Cur-VPDP and PDCP-NP, respectively.

The ability of forming tumor spheroids for a type of cell line is highly correlated with the proportion of CSCs (Gao et al., 2012). Therefore, after treatment with various formulations for three days, the primary glioma spheroids were then further dissociated and the ability of forming tumor spheroids for these dissociated cells was detected by 3D culture. As shown in Figure S8(B&D), a few dense glioma spheroids were formed on the 10th day for group treated with PBS or the combinational Cur/DOX solution or DOX-VPDP, whereas only few of glioma spheroids were observed in groups treated with Cur-VPDP or PDCP-NP. This can suggest that the relapsing ability of tumor spheroid-derived cells was significantly inhibited in groups. Meanwhile, the developed secondary spheroids were further digested into single cells and their cell viabilities were investigated. As expected, the cell viability in the group treated with PDCP-NP was twofold lower than that of the Cur-VPDP-treated group (Figure S8(E)), despite having similar inhibition on the formation of the secondary spheroids. These results might be due to the fact that the combination therapy of DOX and Cur in form of PDCP-NP may not only inhibit the rapid proliferation of the differentiated cells but also enhance the apoptosis of glioma stem cells (Wang et al., 2013).

#### 3.6.2. Inhibitory effect of PDCP-NP on CSCs *in vitro*

**Figure 4.**
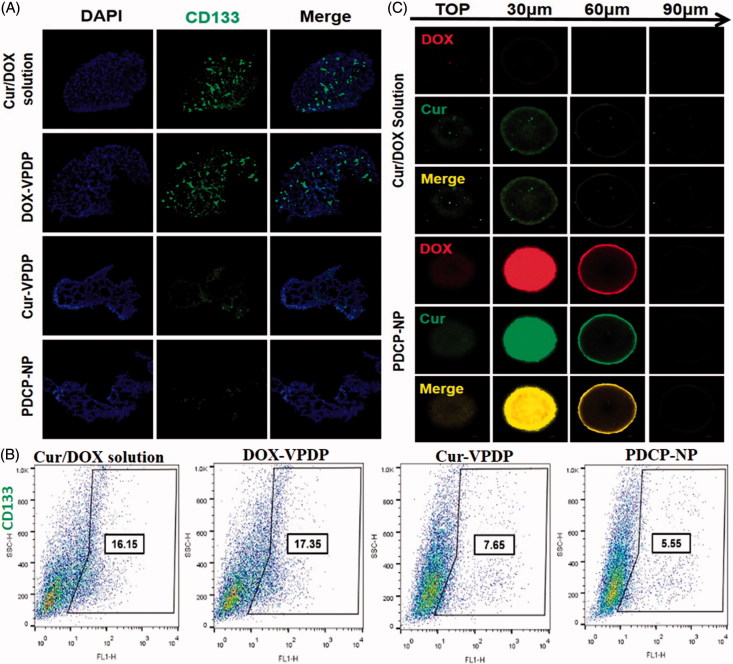
(A) Immunofluorescence cell staining and the (B) proportion of CD133 cells in C6 mammosphere cells after three days of treatment with different formulations. (C) The uptake of Cur/DOX solution and PDCP-NP with equivalent Cur concentration (2 μg/mL) by C6 tumor spheroids at 8 h. Original magnification: 200×.

CD133-positive cells from tumors are responsible for glioma spheroids formation and the CD133-positive sphere cells showed CSC-like characteristics, such as self-renewal, proliferation, and differentiation capabilities (Krause et al., 2017). In order to evaluate whether PDCP-NP could inhibit CSCs *in vitro*, immunofluorescence cell staining was first used to analyze the expression of the CSC-like marker, CD133^+^ inside glioma spheroids after treated with different formulations. As shown in Figure 4, the glioma spheroids treated with Cur-VPDP or PDCP-NP exhibited a dramatically decreased anti-CD_133_ fluorescence in comparison to the groups treated with PBS or the combinational Cur/DOX solution or even DOX-VPDP (Figure 4(A)), indicating an obvious inhibition of CSCs in these groups. Moreover, the percentage of CD133^+^ inside the glioma spheroids was further quantified by flow cytometry. As depicted in Figure 4(B), the percentage of CD133-positive cells inside the glioma spheroids treated with DOX-VPDP attained a value of 17.35%, which was slightly higher than that of control group. Comparing to the PBS treatment, there was only a slight decrease in the percentage of CSCs (16.15%) in glioma spheroids treated with the combinational Cur/DOX solution. But the proportions of CD133 inside the glioma spheroids were significantly diminished to 5.55% and 7.65% after treatment with PDCP-NP and Cur-VPDP, respectively. These results were in correlation with the formation ability of the secondary glioma spheroids after treatment.

#### 3.6.3. The enhanced penetration of PDCP-NP against glioma spheroids

Tumor spheroids can reflect some unique properties similar to those of practical tumors in patients, including a multi-cellular architecture, insufficient provision of oxygen and nutrition in the internal region and an extracellular matrix barrier (Gao et al., 2012, 2014).

In this study, the penetration ability of various NPs into 3D multicellular tumor spheroids was determined by CLSM. CLSM images of C6 glioma spheroids were taken (Figure 4(C)) from their top surfaces to the deeper layers after 8 h incubation with free Cur/DOX, DOX-NP, Cur-NP, and PDCP-NP. A weak red and green fluorescence was seen in the group treated with the combinational Cur/DOX solution, indicating a poor penetration of Cur and DOX into the glioma spheroids, while a remarkable fluorescence of DOX and Cur were observed at the deeper layer of the glioma spheroids treated with PDCP-NP. Moreover, the green fluorescence of curcumin and the red fluorescence of DOX overlapped perfectly in the PDCP-NP group, which also suggests a simultaneous delivery of Cur and DOX to the same site of glioma spheroids. The superior tumor penetrating ability of VES-g-ε-PLL nanoparticle was demonstrated in our previous study (Xu et al., 2016) and its penetrating ability was not compromised after coating with the γ-PGA-Dopa polymer in this study.

### 3.7. *In vivo* anti-tumor efficacy and animal survival

**Figure 5.**
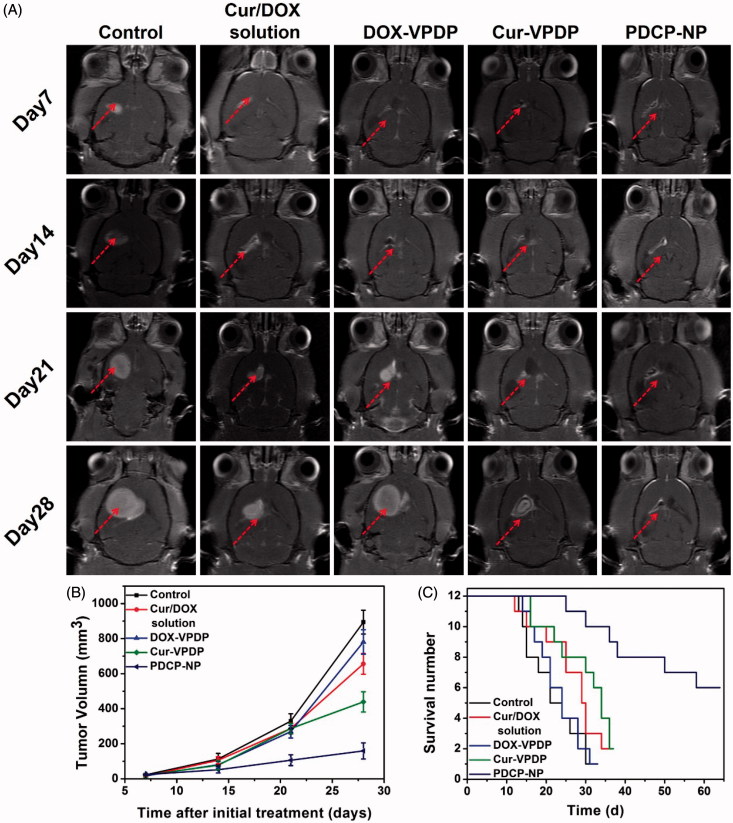
(A) MRI images of the brains of glioma rats, (B) the calculated tumor volume, and (C) the survival curves of glioma-bearing rats after different treatments.

To investigate the efficiency of PDCP-NP on growth inhibition of glioma *in vivo*, UTMD was exploited to temporally open the BBB to deliver PDCP-NP into brain tumors. Tumor progression was longitudinally monitored by an enhanced T1-weighted MRI and tumor volume was calculated accordingly by MRI imaging. As shown in Figure 5(A,B), tumor lesions were easily observed in the brain MRI images of rats from each group and their volumes were increasingly growing with time in rat models treated with the combinational Cur/DOX solution or DOX-VPDP, exhibiting a slight inhibition of tumor growth *in vivo*. Tumor volumes in glioma-bearing rats treated with saline or DOX-VPDP increased from 23.15 ± 5.11 mm^3^ and 18.24 ± 8.46 mm^3^ on day 7 to 894.45 ± 68.52 mm^3^ and 781.40 ± 70.18 mm^3^ on day 28, respectively. At the same time, tumors of rats treated with the combinational Cur/DOX solution and Cur-VPDP grew to 625.73 ± 66.58 and 421.95 ± 43.87 mm^3^ on day 28, respectively. However, an obvious inhibition of tumor growth was observed after treatment with PDCP-NP. Tumor volumes in glioma-bearing rats treated with only PDCP-NP grew to 159.18 ± 14.92 mm^3^ on day 28. Besides, rat survival after treatment with the PDCP-NP (64.5 ± 6.3 days) was significantly prolonged in comparison to the control group (31.4 ± 2.1 days, *p* < .001), the combinational Cur/DOX solution group (36.3 ± 3.4 days, *p* < .001), the DOX-VPDP group (33.5 ± 2.1 days, *p* < .001) and the Cur-VPDP group (38.7 ± 2.8 days, *p* < .001) (Figure 5(C)).

### 3.8. *In vivo* drug distribution

*In vivo* distribution of PDCP-NP was investigated by *ex vivo* fluorescent imaging of the isolated organs including the brain, heart, liver, spleen, lung, and kidney, as well as the tumors 2 h post-injection. As shown in Figure S9(A) (supplementary information), only a weak Cur fluorescence was exhibited in the kidney 2 h after treatment with the combinational Cur/DOX solution and there was merely any distribution of the drug in other organs. This reflected that the free Cur or DOX was quickly eliminated through the kidney. By contrast, a strong Cur and DOX fluorescence was exhibited in lung, liver and kidney after treatment with PDCP-NP. Apart from that, a marked increase in the distribution of Cur and DOX was also observed in the brain when compared to combinational Cur/DOX solution group. However, there was no obvious fluorescence observed in the spleen for the PDCP-NP treated group and it may be due to the γ-PGA shell avoiding capture by the RES system (StevanovicKovacevic et al., 2011).

Whether the enhanced distribution of PDCP-NP in the brain was associated with its higher tumor distribution was further confirmed by imaging a slice of the glioma. The resultant fluorescent images are shown in Figure S9(B). There was no obvious Cur or DOX fluorescence appearing in the glioma zone of rats treated with the combinational Cur/DOX solution. In contrast, an obvious and strong Cur or DOX fluorescence was observed in tumor tissues of rats administered with PDCP-NP, indicating that PDCP-NP truly facilitated more Cur and DOX molecules to accumulate in the glioma, which was consistent with the above results.

Furthermore, the ratiometric delivery of the combinational Cur and DOX to glioma was also confirmed by detecting their amount in tumor tissues per 100 mg. Two hours after administration of the various formulations, the glioma-bearing rats were sacrificed and glioma tissues were isolated and homogenized for HPCL analysis. Results are presented in Figure S9(C). The amount of Cur and DOX in glioma of rats treated with combinational Cur/DOX solution was only 0.10 ± 0.09 μg and 0.08 ± 0.11 μg per 100 mg tumor tissue, respectively. Moreover, the calculated Cur/DOX ratio in glioma was 1.2:1 for treatment with the combinational Cur/DOX solution, despite having an initial administrated dose ratio of 3:1. By contrast, the amounts of Cur and DOX in glioma of rats treated with PDCP-NP were increased to 1.08 ± 0.21 μg and 0.31 ± 0.12 μg, indicating an effective drug delivery of PDCP-NP to the glioma under the assistance of UTMD. Moreover, the calculated Cur/DOX ratio in tumors treated with PDCP-NP was 3.2:1, which was close to the initial ratio of Cur/DOX (3:1) encapsulated in the nanoparticle carrier. These results indicated that PDCP-NP could affect *in vivo* distribution of their encapsulated cargos and perform ratiometric delivery of the encapsulated Cur/DOX into the glioma.

### 3.9. Inhibition of CSC population in glioma after treatment of PDCP-NP

**Figure 6.**
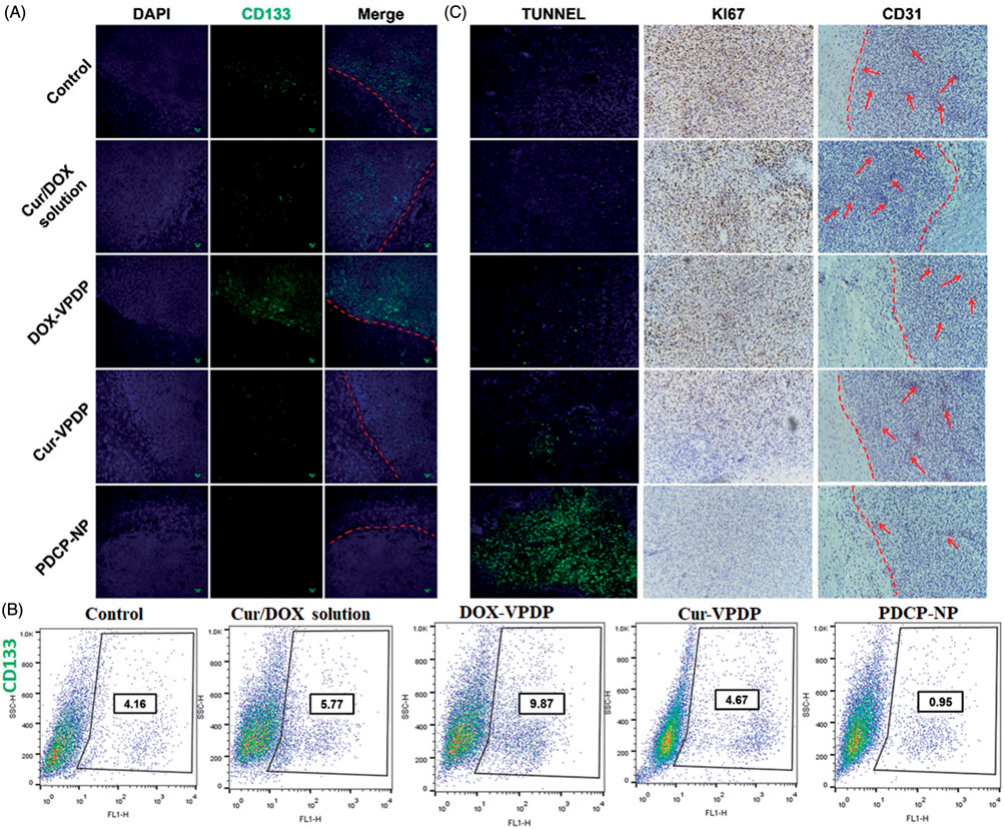
(A) Immunofluorescence tissue staining and (B) the CSCs proportion within tumors after the tumor suppression study. (C) TUNEL assays of C6 glioma sections from rats receiving different therapies on day 28. Nuclei were stained blue while extracellular matrix and cytoplasm were stained red in HE staining. In TUNEL analyses, blue: cell nuclei stained by DAPI. Green: apoptotic cells. Immunohistochemical assays of Ki67, CD31 in tumor tissue. Red arrows highlight the immunohistochemical characteristics. Original magnification: 200×.

To confirm *in vivo* anti-CSC capacity of PDCP-NP, the expression of CD133^+^ in the glioma was further analyzed by immunohistochemical staining. As shown in Figure 6(A), the stem cells marker, CD133^+^ was highly expressed at the boundary between the tumor tissue and the normal tissue in the saline group. Moreover, treatment with the combinational Cur/DOX solution or DOX-VPDP exhibited no obvious inhibition of glioma stem cells, exhibiting a strong fluorescence of CD133^+^ similar to that of the saline control. However, there was an obvious inhibition of glioma stem cells in tumors treated with Cur-VPDP or PDCP-NP as a weaker fluorescence of CD133^+^ was observed at the boarder of the glioma. Moreover, the expression of CD133^+^ in the glioma treated with PDCP-NP was significantly lower than that of the Cur-VPDP group. Alternatively, the glioma stem cell population after treatment was further quantified by flow cytometry. As shown in Figure 6(B), glioma stem cells accounted for 4.16% of the total cells inside the glioma of the control group (PBS treatment) and its proportion was increased to 5.77% and 9.49% after treatment with the combinational Cur/DOX solution and DOX-VPDP, respectively. In contrast, the proportion of CD133^+^-positive cells in glioma decreased to 0.95% after treatment with PDCP-NP, indicating a significant inhibition of CSCs in glioma. The stronger inhibition of CSCs might be associated with the ratiometric delivery of Cur/DOX through the pH-sensitive core–shell nanoparticles.

### 3.10. Immunohistochemistry staining of glioma

Cellular apoptosis inside the glioma after different treatments was first detected by TUNEL assay and the results are shown in Figure 6(C). Most of the cells inside the tumor zone were undergoing apoptosis after treatment with PDCP-NP, while only few of the apoptotic cells were observed in the groups of the combinational Cur/DOX solution, DOX-VPDP or Cur-VPDP. These apoptotic results also confirmed that treatment with the PDCP-NP was most effective in inducing tumor cell apoptosis, which correlates to the inhibition of CSCs. Alternatively, cellular proliferation can also be reflected by Ki-67 expression (AhmedRashed et al., 2016). As shown in Figure S9(C), an obvious inhibition of cellular proliferation was also exhibited in the group of PDCP-NP, but most of the tumor cells in the other groups were still active to proliferation. Angiogenesis was highly correlated with the expression of CD31 at the tumor site. Moreover, the expression of CD31 is positively related to tumor invasion and chemotherapy resistance (Gong et al., 2013). In this study, the anti-angiogenesis ability of PDCP-NP was evaluated by staining CD31, a marker for neo-vasculature. A high level of CD 31 expression was observed in the tumor without any treatment (control group), but the expression of CD 31 significantly decreased after treatment with PDCP-NP. These results demonstrated that the PDCP-NP also displayed effective anti-angiogenesis, which might be an important reason why PDCP-NP produced the best anti-tumor effect.

### 3.11. Toxicity evaluation

To investigate the toxicity of PDCP-NP against normal tissues, the main organs from healthy rats were collected and sliced for (H&E) staining after injection of PDCP-NP. As shown in Figure S10, no indication of damage was observed in these organs after treatment, suggesting that treatments by intratumor administration did not cause systemic toxicity. Rate administrated with Cur/DOX solution, DOX-VPDP Cur-VPDP or PDCP-NP, demonstrated neither noticeable organ damage nor inflammation lesions in the heart, liver, spleen, lung, and kidney, indicating that PDCP-NP had no significant toxicity to the treated rats. Both the relatively low dose and the co-delivery of the two drugs via a nanoparticulate drug delivery system used in this study were of advantages to reducing side effects.

In addition, normal brain tissue adjacent to the glioma was also detected via HE staining and the results are shown in Figure S11. No cytotoxicity against normal brain tissues was observed with the use of for PDCP-NP, indicating that the latter is relatively safe to be used.

## 4. Discussion

Combination chemotherapy regimens are typically developed for cancer treatment by establishing the recommended dose of one drug and then adding subsequent agents to the mixture at incremental doses until the aggregate cytotoxicity effects become dose-limiting (Mayer & Janoff, 2007). This is done with the assumption that maximum therapeutic activity can be achieved with maximum dose intensity of all drugs in the mixture and may overlook the possibility that more subtle concentration-dependent drug interactions could achieve synergistic outcomes or in some instances, antagonism. Not only is synergy dependent on the distinct pharmacological actions of each drug but individual agents in a conventional anticancer drug combination are distributed and eliminated independently. This could turn out to be essential if the extent of synergy depended on the ratio and concentrations of the drugs in the mixture. A ratiometric approach to drug delivery could overcome these problems (Meng et al., 2015). Co-delivery of curcumin and DOX has been used to improve cancer therapy as explained in few literatures (Wang et al., 2013; ZhaoChen et al., 2015; Gu et al., 2016; Zhang et al., 2016). For example, Gu et al. used PEG-linked amphiphilic copolymer and TPGS_1000_ assembled micelles to co-encapsulate curcumin and DOX (Wang et al., 2013). Zhang et al. designed a PEG-DOX-Cur prodrug nanoparticle for simultaneous delivery of DOX and curcumin (Cur) as a combination therapy to treat cancer (Zhang et al., 2016). Although they have shown improved anticancer efficacy against different tumors, none of them were able to simultaneously delivery curcumin and DOX at a predetermined ratio to exert optimum anti-tumor effect. For most of them, the loaded DOX or curcumin was through chemical bonds or the loaded curcumin and DOX were all in a single micelle. In this study, the curcumin was first encapsulated into cationic nanoparticles of VES-g-ε-PLL to prepare for the Cur-loaded nanoparticles (Cur-NPs). Second, the DOX hydrochlorate was encapsulated in the γ-PGA outer shell through electrostatic interaction. Unlike the synthetic polymers, ε-PLL and γ-PGA are the most important naturally occurring homo-polypeptides, and known to be fully biocompatible, biodegradable, and nontoxic. In our previous study, VES-g-ε-PLL showed negligible toxicity toward blood red cells and higher graft ratio of VES would further increase the compatibility of the grafted polymers. Regarding the γ-PGA-Dopa polymer, its application tissue engineering, drug delivery and biosensor have been widely reported, and γ-PGA-Dopa polymer exhibited considerable biocompatibility in those studies. Considering the good biocompatibility and ideal physicochemical characteristics, we could easily control Cur/DOX ratio by adding the desired amounts of drugs at different steps, and used for synergistic combination therapy.

Cancer stem cells, also called tumor-initiating cells or stem-like cancer cells are associated with glioma metastasis and recurrence after treatments (Singh & Settleman, 2010). Our study first investigated the combination cytotoxicity of Cur/DOX against not only C6 adherent cells but also C6 stem cells and screening of the optimum ratio of Cur/DOX was also performed. It has been documented that CSCs are more resistant to drug treatment than bulk cancer cells (Gersey et al., 2017). Although several literatures have investigated and reported the therapeutic effects of curcumin against glioma heterogeneous cells (Miller et al., 2012; LiaoWang et al., 2015; Sobolewski et al., 2015), a concentration-dependent decrease in stem cell viability was observed in Figure S7 and the cytotoxicity of Cur/DOX solution against C6 adherent cells was significantly increased as the DOX proportion increased. The dose ratio of Cur/DOX at 3:1 was found to be the most effective in inhibiting the heterogeneous cells in tumor tissues. Curcumin and DOX were also successfully loaded in VPDP-NP at the weight ratio close to 3:1.

After intravenous administration, the drug-loaded nanoparticles can come into contact with blood components, including plasma protein. Adsorption of plasma proteins on the surface of the nanoparticles or an interaction between the plasma protein and the encapsulated drug would result in the breakdown of the drug-loaded nanoparticles or premature drug leakage (Patel & Tannock, 2009). In this study, the stability of PDCP-NP against the plasma protein was detected by measuring the particle size and zeta potential (Figure 2(C)). The stability of PDCP-NP contributed to the stabilization of the Cur/DOX ratio in physical condition (Figure 2(D)). Furthermore, PDCP-NP performed a synchronous release of its encapsulated curcumin and DOX for a long time without obvious drug leakage in a physiologic medium, while a rapid release of curcumin and DOX was observed in a pH 5.0 medium. The pH 5.0 medium also promoted a rapid escape of the loaded drugs from the endosomes. Therefore, it is worthy to mention that these factors play an important role in the ratiometric delivery of Cur/DOX.

Penetrating the dense tumor tissues has been an insurmountable barrier because of the high tumor cell density and an abnormal extracellular matrix (Grantab et al., 2006; Qi et al., 2017). The strong penetrating ability and the growth inhibition of VES-g-ε-PLL micelles against C6 spheroids was reported in our previous report (Xu et al., 2017). Its penetration ability was not compromised after the latter was grafted with γ-PGA-Dopa. The penetrating ability of PDCP-NP was attributed to the following reason. γ-PGA can increase the intracellular uptake of cancer cells (Chaffer et al., 2011) and thus improving the efficiency of drug delivery. These may also explain the relative high intracellular drug content both *in vitro* (glioma spheroid) and *in vivo* (solid tumor). Notably, the intracellular weight ratio of Cur and DOX was similar to the administered ratio. It was suspected that the ratiometric delivery of curcumin and DOX were attributed to the following aspects. First, γ-PGA is an anionic polymer with the properties of nontoxicity, biodegradability, biocompatibility, and nonimmunogenicity, which all together assisted to the stability of PDCP-NP *in vitro* and *in vivo*. Second, γ-PGA contributed to the intracellular uptake of cancer cells, thus improving the efficiency of drug delivery. The synchronous release of curcumin and DOX in pH 7.4 and pH 5.0 media was therefore developed.

In this study, tumor cell spheroids were used to enrich CSCs. The therapeutic effect of the nanoparticles on the self-renewal ability of stem cell was examined by evaluating the formation of glioma spheroids. It was shown that Cur-VPDP and PDCP-NP reduced both the number and size of primary glioma spheroids formed using the C6 cell line. This observation is most apparent after treatment with PDCP-NP. Our delivery system also reduced the stemness of the C6 glioma spheroid cells by PDCP-NP, as indicated by a reduction of the stemness marker, CD133. However, DOX-VPDP induced the enrichment of CSCs in tumor cells by stimulating the non-stem cells that have converted to a stem-like state (Shi et al., 2014). In addition, the CSC markers were significantly deceased after Cur-VPDP and PDCP-NP treatment *in vitro*, while the PDCP-NP treatment group exhibited much stronger anti-tumor effect *in vivo* compared to the Cur-NP treatment group. These evidence indicated that non-CSCs in the tumor can spontaneously and stochastically turn into CSCs *de novo*. Accordingly, co-delivery of curcumin and DOX showed a more superior therapeutic efficiency than administration of single drug loaded nanoparticles (Shi et al., 2014). Therefore, our drug delivery system was designed specifically to eliminate heterogeneous tumor cells, which showed promising efficacy both in cell and animal models (Xu et al., 2017).

## 5. Conclusions

In the study, a pH-sensitive core–shell micelles were constructed by using the VES-g-ε-PLL and γ-PGA-Dopa as materials. In this micelle, vitamin E succinate was served as a hydrophobic moiety to encapsulate the poorly water-soluble model drug, curcumin and the outer shell polymer γ-PGA was used for loading the water-soluble model drug, DOX. We proved that the co-delivery system PDCP-NP can carry on a precise ratiometric delivery of CUR and DOX to heterogeneous tumor cells of glioma *in vitro* and *in vivo*. The unique penetration ability of PDCP-NP resulted in an excellent cytotoxicity and growth inhibition against tumor spheroids. Moreover, the enhanced inhibition of tumor stem cells, the slower growth and the suppressed invasion of glioma *in vivo* were also exhibited after treatment with PDCP-NP combined with UTND. Overall, the synergistic therapeutic effects between Cur and the DOX have been indicated in this study by exploiting the novel nanoplatform of VPDP-NP.

## Supplementary Material

Supplemental Material
