# Supplementary material for: Ratiometric delivery of two therapeutic candidates with inherently dissimilar physicochemical property through pH-sensitive core–shell nanoparticles targeting the heterogeneous tumor cells of glioma
Source: Drug Deliv. 2018 Jun 5;25(1):1302–18. doi: 10.1080/10717544.2018.1474974 (PMC6060705; doi:10.1080/10717544.2018.1474974)
Supplement: Supplemental Material [file IDRD_A_1474974_SM0162.docx]

**Supplementary information**

**1 Method**

**1.1 Synthesis of γ-PGA-Dopa and VES-g-**ε**-PLL copolymer**

The synthesis method of VES-g-ε-PLL was reported in our previous research [[1](#_ENREF_1)].γ-PGA-Dopa was synthesized by condensation reaction between carboxyl of γ-PGA and amino groups of dopamine in presence of the carbodiimide (EDC). Briefly, dopamine (0.5g) was dissolved in distilled water of 10mL, and the carboxyl of γ-PGA in distilled water was activated in presence of EDC and NHS (molar ratio of VES, EDC and NHS, 1:1:1). The activated γ-PGA solution was added dropwise to dopamine solution under stirring at room temperature. After reaction for 12h, the whole reaction solution was dialyzed against distilled water for 48h using dialysis membrane with cutoff molecular weight of 3500Da, and then lyophilized.

**1.2 In vitro internalization of** **PDCP-NP.**

Internalization of PDCP-NP by tumor cells was further analyzed by TEM. First, C6 cells were seeded onto 6-well culture plates at a density of 2×10^4^ per well and cultured in complete DMEM for 24 h. And then, the isolated cells were incubated with fresh complete DMEM containing PDCP-NP (75μg/ml). After 10min, 1h or 8h incubation, C6 cells were fixed with 2.5% glutaraldehyde and 0.1 M sodium cacodylate for 1 h. The samples were then rinsed with sodium cacodylate, followed by a second fixing with 1% osmium tetroxide for 1 h. The samples were dehydrated by rinsing with 50%, 70%, 95%, and 100% ethanol. The samples were impregnated overnight in a 1:1 mixture of acetone and Epon 812 at room temperature. Then the samples were embedded in Epon 812 resin at 60°C. The embedded samples were cut in sections of 50-70 nm, using a LeicaEMUC6 microtome (Leica, Groot Bijgaarden, Belgium). Sections were then transferred to a copper grid coated with a carbon film. The samples were lightly stained with 0.5% uranyl acetate and lead citrate solution.

**2 Results**

The successful reaction of γ-PGA and dopamine was confirmed by ^1^H-NMR and FT-IR. For example, ^1^H-NMR and FT-IR spectra of γ-PGA-Dopa copolymer were shown in Fig.S1A. The peaks specific to γ-PGA appeared between 1.1ppm and 4.0ppm and the peaks at 3.56ppm, 1.08ppm and 2.26-1.84ppm were attributed to methyne (CO-CH (NH_2_)-), terminal methylene (CH_2_CO-) and middle methylene (-CH_2_-CH_2_-) groups of γ-PGA backbone, respectively. Characteristic peaks of Dopa appeared at 2.74-3.17ppm and 6.64-6.78ppm. In contrast, ^1^H-NMR spectrum of γ-PGA-Dopa copolymer showed groups of peaks at 6.63-6.79ppm and 2.78-3.12ppm, which were corresponding to dopamine unites, and peaks at 1.84ppm, 2.26ppm and 1.08ppm corresponded to both of middle methylene, which was attributed to γ-PGA unites. Besides, for γ-PGA-Dopa polymer, a new peak assigned to methyne of γ-PGA shifted from 3.56ppm to 3.77ppm, indicating a part of amine groups were acylated. Fig.1B showed the FT-IR spectra of γ-PGA, dopamine and γ-PGA-Dopa. The γ-PGA peaks located at 1645, 1588, 1120cm^-1^ were assigned to C=O stretching vibration, -CONH- bond vibration and C-N bond vibration, respectively. Dopamine peaks located at 2930 cm^-1^ and 1504 cm^-1^ were assigned to C-H vibration and C-N ring stretching, whereas the absorption peaks located at 1606 and 1278 cm^-1^ were attributed to C=C ring stretching and C-O stretching vibrations, respectively. Moreover, the FT-IR spectrum of γ-PGA-Dopa displayed groups of peaks at 1639，1583 and 1118 cm^-1^ which were also showed in γ-PGA. The peaks at 3073cm^-1^(O-H stretching vibration) and 1288cm^-1^ (C-O stretching vibrations) also appeared in γ-PGA-Dopa sample. These results together demonstrated that Dopa was successfully grafted onto γ-PGA.


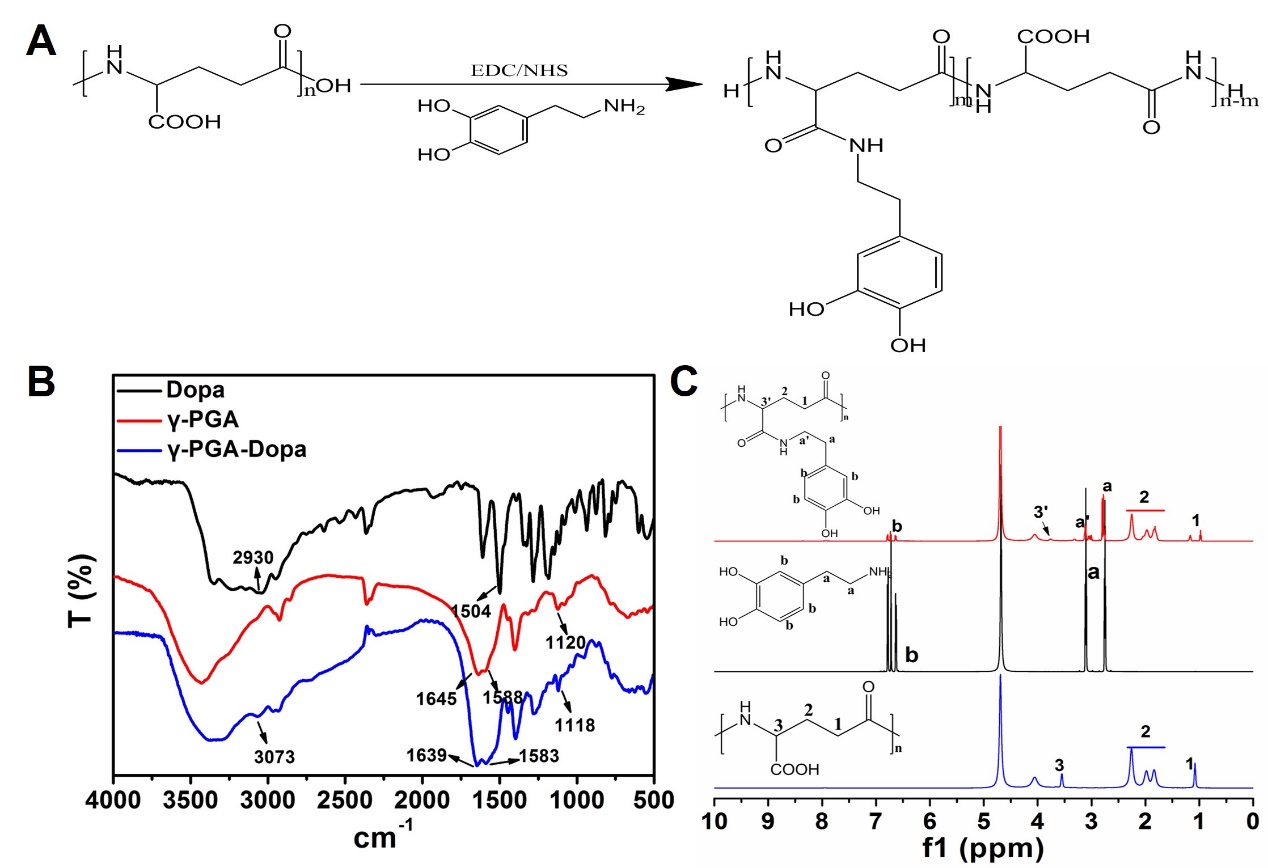


Fig S1 (A)The synthetic routes of the γ-PGA-Dopa copolymer (B) FT-IR and (C) ^1^H-NMR spectra of dopamine, γ-PGA and γ-PGA-Dopa polymer (Samples dissolved in D_2_O for ^1^H-NMR) The number of 1, 2, 3… and alphabet of a, b, c,… in figure 1 were referred for hydrogen in different chemical environment in Dopa and γ-PGA, respectively.


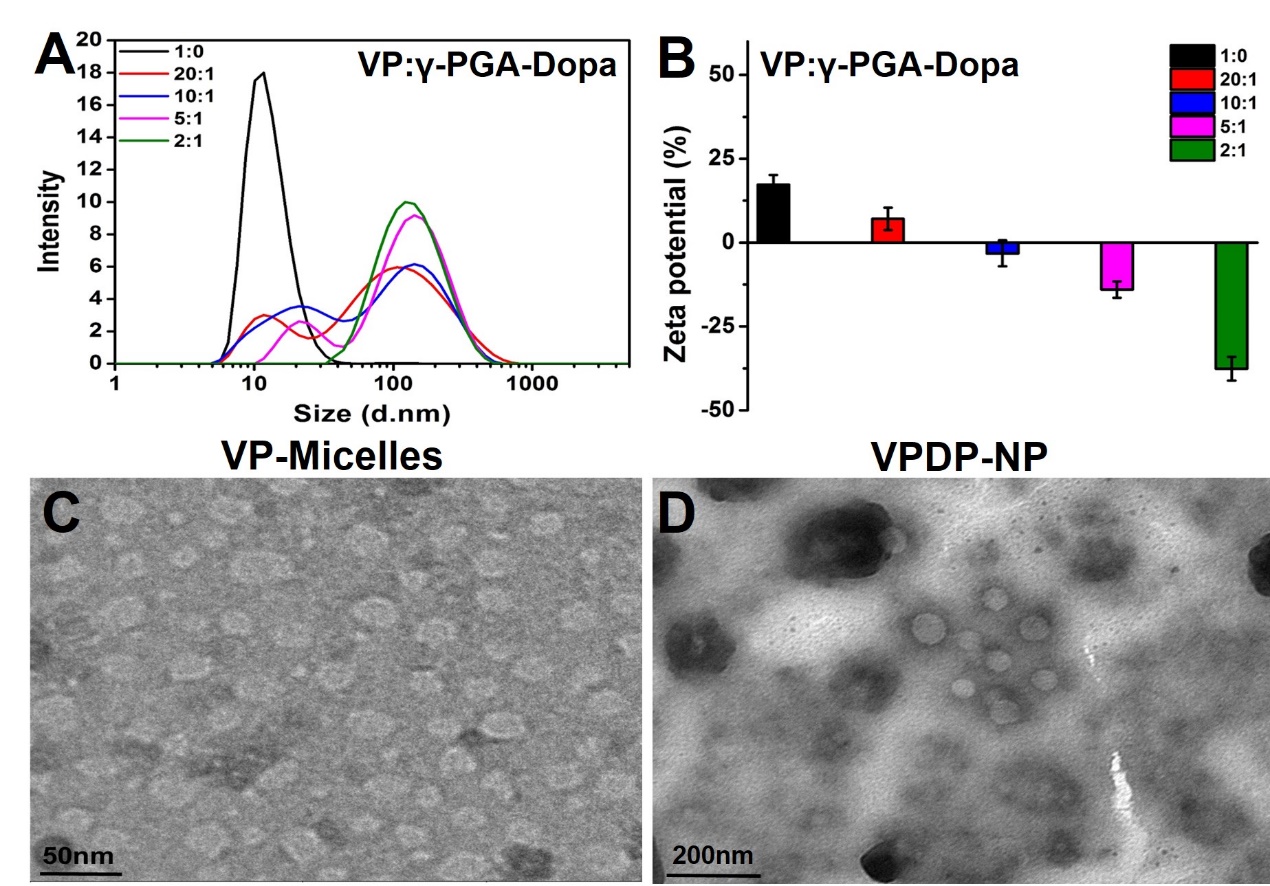


Fig S2. The particles size distributions (A) and Zeta potential (B) of VES-g-ε-PLL nanoparticle (VP) and VPDP-nanoparticle, and TEM graphics of nanoparticles of VP nanoparticle (C) and VPDP nanoparticle at VP/γ-PGA-Dopa of 2:1 (D)

**
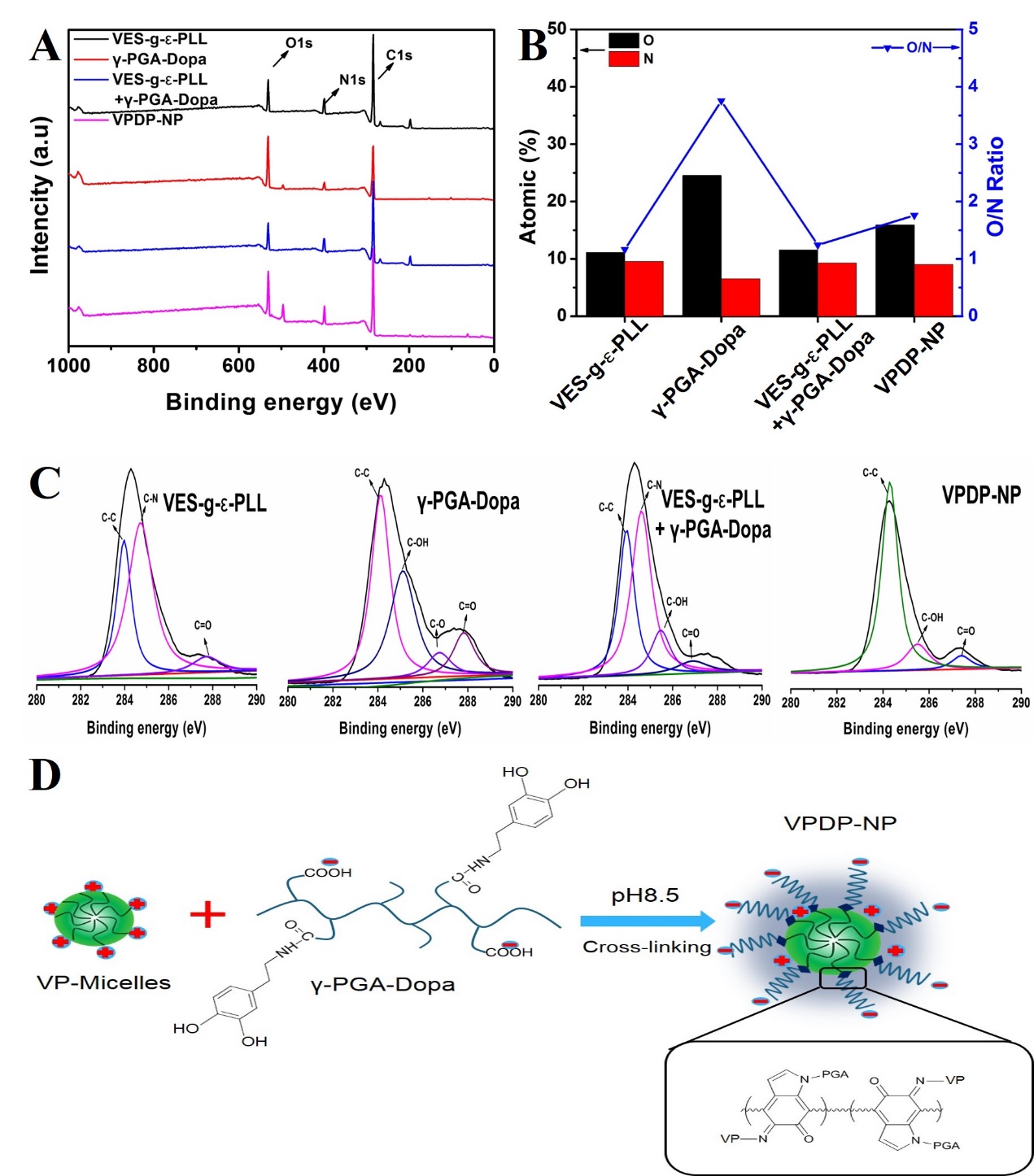
**

Fig S3 (A) XPS spectra of different nanoparticles, (B) the corresponding composition and the element ratio of N/O in these formulations (C) C1s peaks of XPS spectra in different nanoparticles (D) Schematic diagram of surface modification of VP micelles using γ-PGA-Dopa


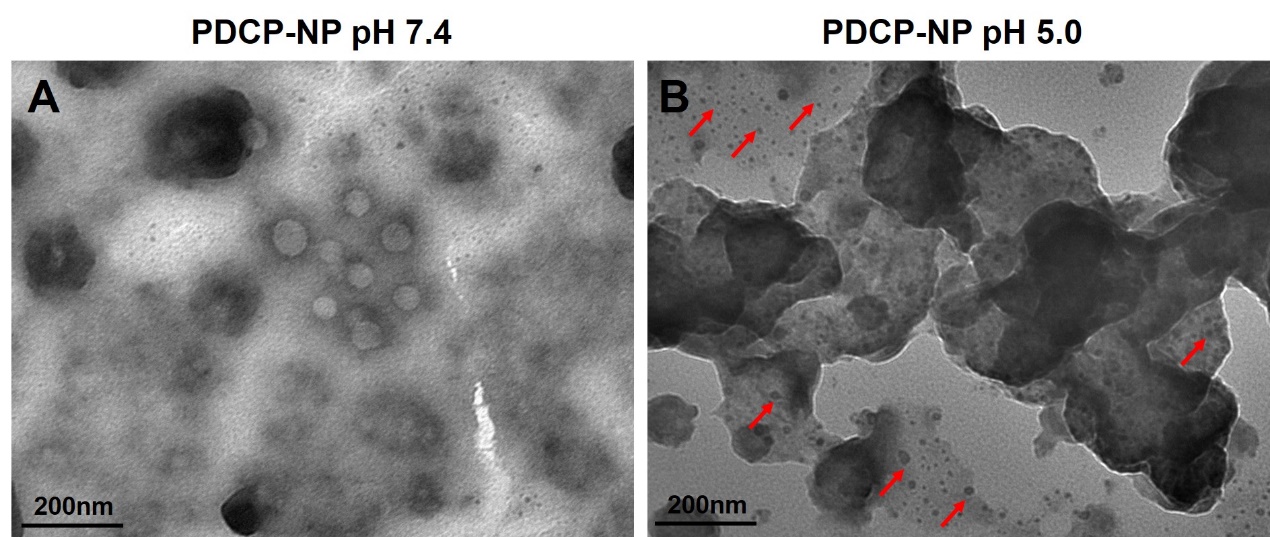


Fig S4 TEM micrographics of drug-loaded nanoparticles of VPDP-NP in pH7.4 PBS and pH5.0 acetate buffer.

**
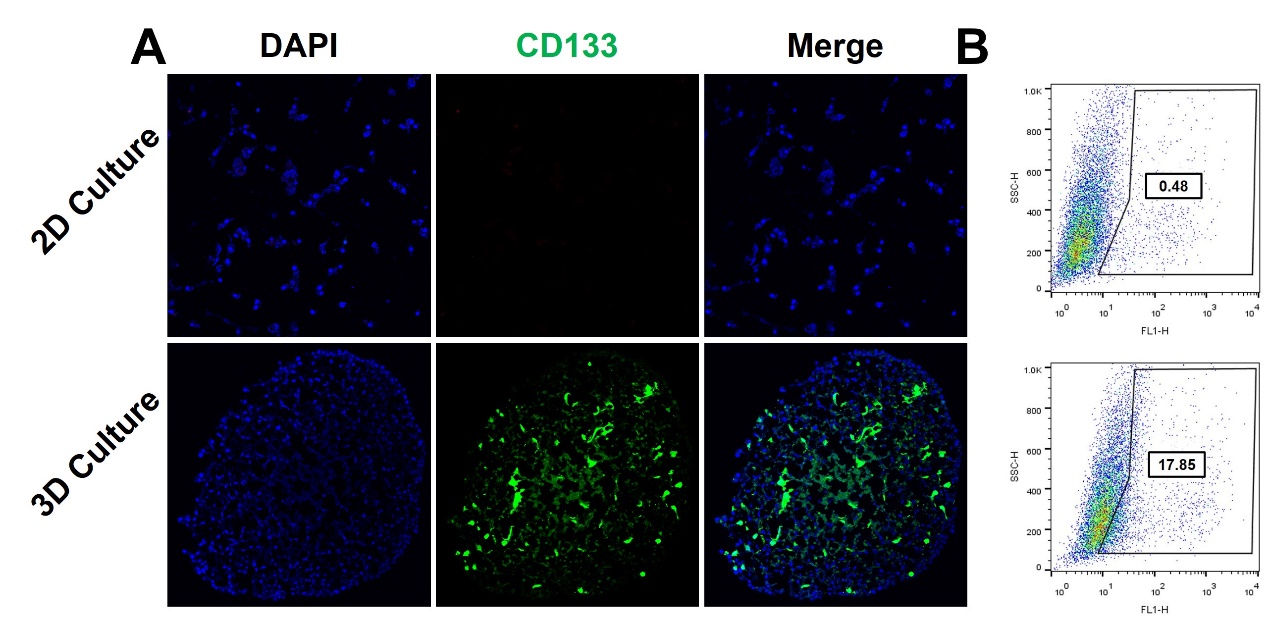
**

Fig S5 (A) Immunofluorescence cell staining and (B) flow cytometry of CD133^+^ cells in C6 glioma spheroid. Original magnification: 200×


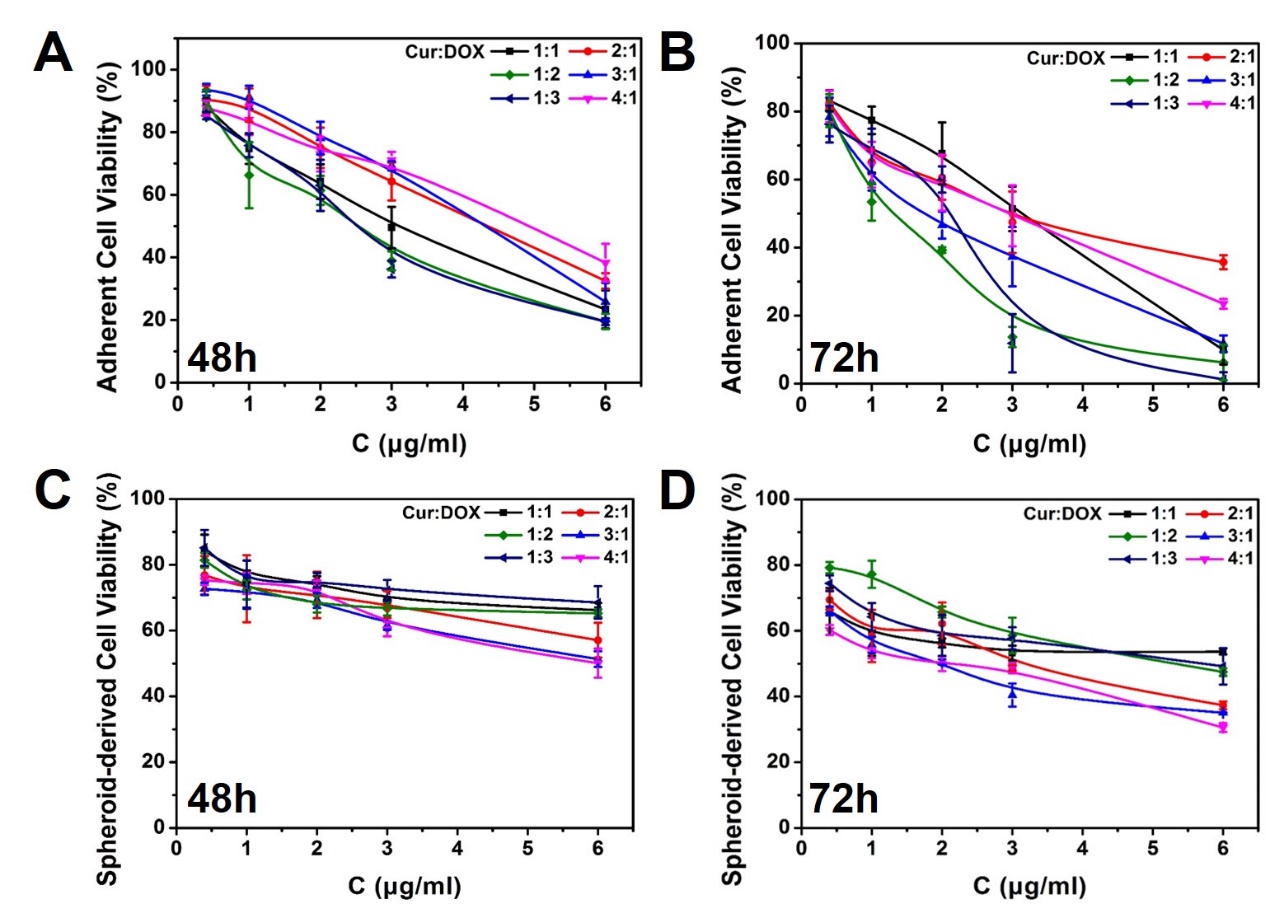


Fig S6 C6 adherent cells or glioma spheroids-derived C6 cells were exposed to different concentrations (0.4–6μg/ml) of Cur/DOX solution with various dose ratio for 48 hours(A) and 72 hours(B), respectively. glioma spheroids-derived C6 cells were exposed to different concentrations (0.4–6μg/ml) of Cur/DOX solution with different ratio for 48 hours(A) and 72 hours(B), respectively.

**
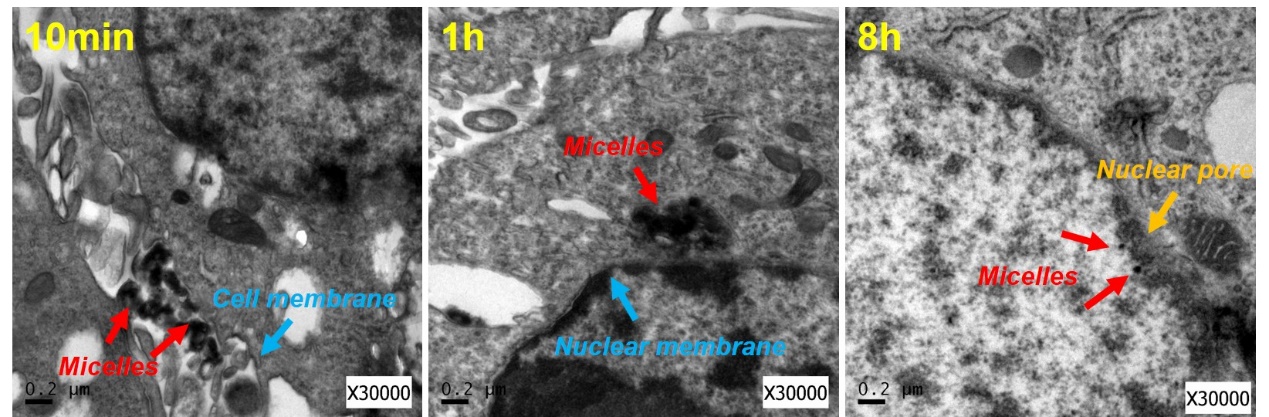
**

Fig S7 TEM images of ultrathin sections of glioma tumor derived C6 cells incubated with PDCP-NP for different time, showing that nanoparticales enters the nucleus via nucleopores and the nuclear localization of the naked VES-g-PLL nanoparticles.

**
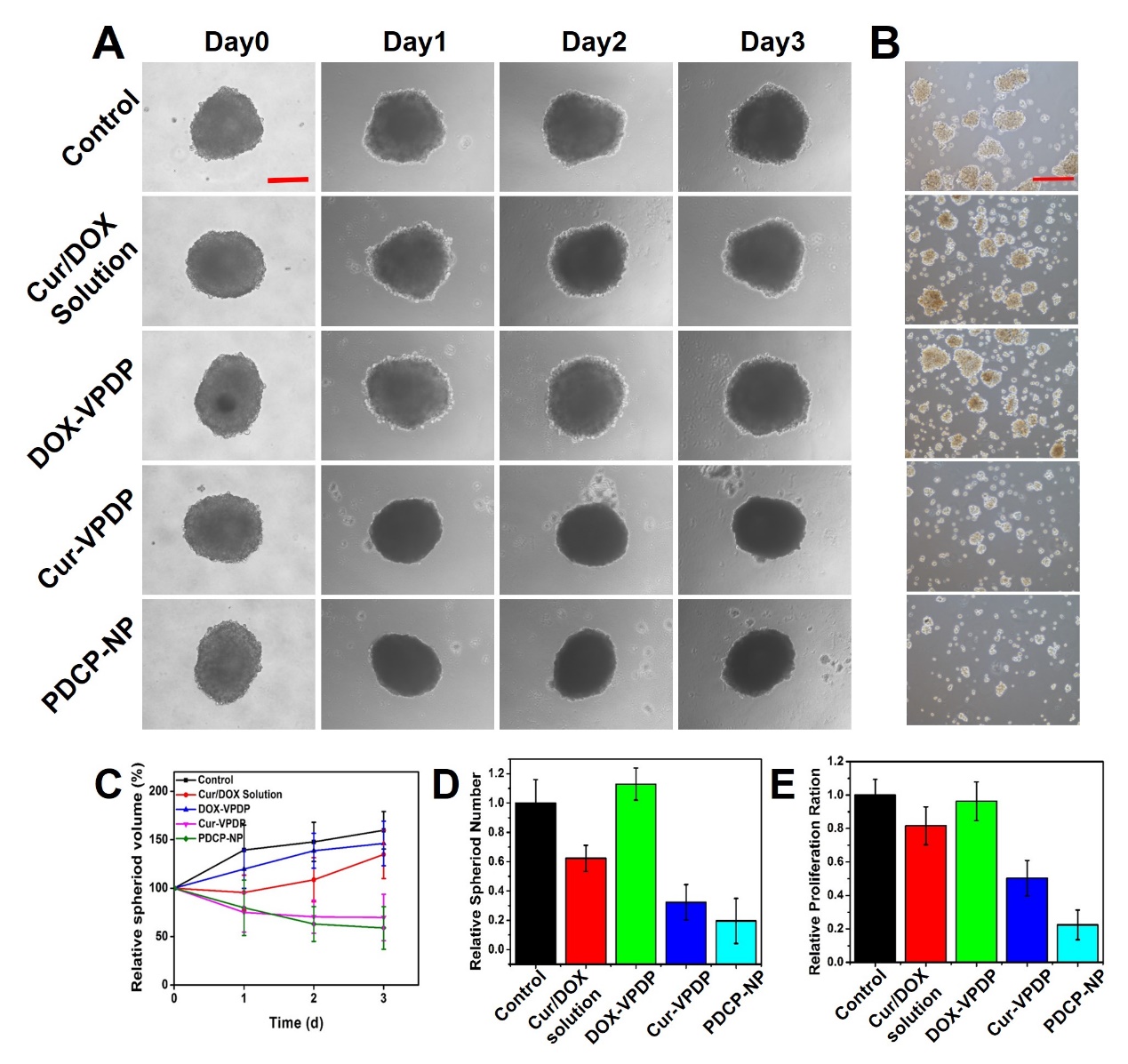
**

Fig S8 (A) The representative C6 glioma spheroids images observed by using an inverted microscope on day 1, day 2 and day 3 after treated with Cur/DOX solution, DOX-VPDP, Cur-VPDP or PDCP-NP. Glioma spheroids without any drug treatment served as control. (C)The volume changes of glioma spheroids in each group after different micelles formulations treatments. Glioma spheroids without any drug treatment served as control. The spheroid formation (B), the number of spheres (D) and the percentage of viable cells (E) was measured by the CCK-8 assay after treatment with different formulations for 3 days. Original magnification: 200×.


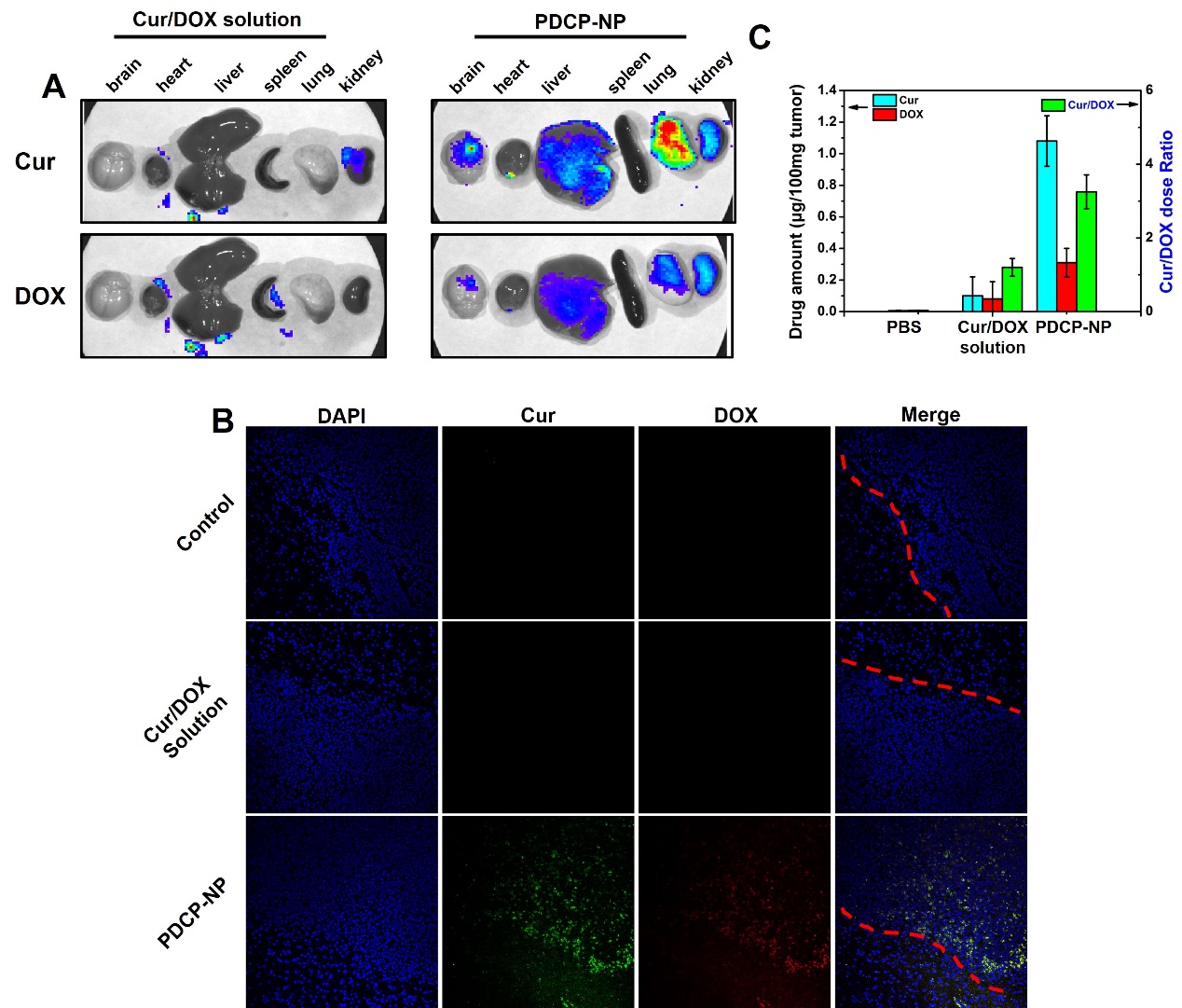


Fig S9 (A) ex vivo fluorescence images of dissected organs from glioma-bearing rats at 2 h after treatment with various formulations, (B) The fluorescent image of the tumor slice (Red fluorescence emitted by DOX; Green fluorescence emitted by Cur; Red line indicated the border of glioma; Red arrow indicated the glioma zone; Original magnification: 200×), and (C) The amount of Cur and DOX in glioma and their corresponding dose ratio after different treatments.


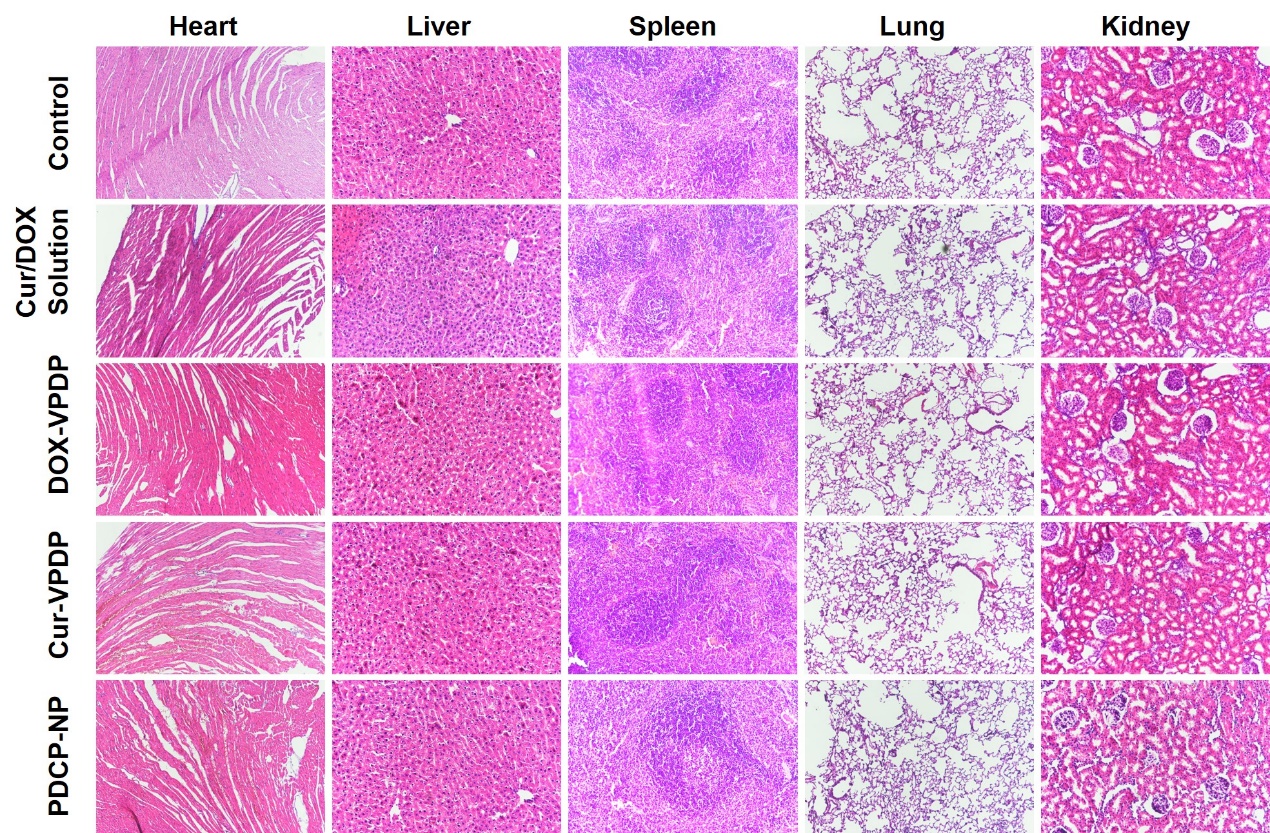


Fig S10 Histological staining of organs from rats treated with different formulations. Original magnification: 200×.

**
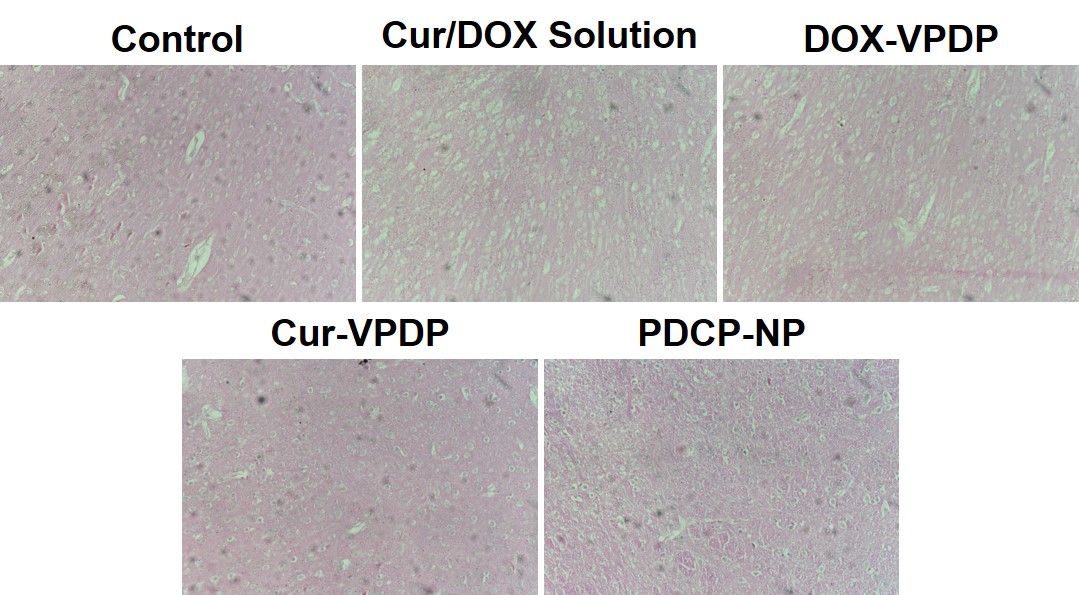
**

Fig S11 HE staining of normal brain adjacent to glioma after different treatments. Original magnification: 200×

**Table S1** Characteristics of the different nanoparticles

| Formulations | D_h_ (nm) | Zeta potential | DL (%) | EE (%) |
| --- | --- | --- | --- | --- |
|  |  | (mV) |  |  |
| VP micelles | 19.34±5.94 | 17.3±2.87 | / | / |
| VPDP-NP | 122.4±14.21 | -37.6±6.12 | / | / |
| Cur-VPDP | 141.77±12.43 | -39.1±5.83 | 4.78±0.35 | 84.14±0.54 |
| DOX-VPDP | 118.32±10.19 | -28.45±7.32 | 3.64±0.59 | 87.34±6.87 |
| PDCP-NP | 160.8±8.64 | -30.6±4.98 | 4.46±0.87(Cur); 1.47±0.49(DOX) | 85.07±2.86 Cur);  93.64±4.97(DOX) |

**References**

[1] Xu HL, Mao KL, Lu CT, Fan ZL, Yang JJ, Xu J, et al. Biomaterials. 2016;107:44-60.

[2] Kim WJ, Kim BS, Cho YD, Yoon WJ, Baek JH, Woo KM, et al. Fibroin particle-supported cationic lipid layers for highly efficient intracellular protein delivery. Biomaterials. 2017;122:154-62.

[3] Ma D, Lin QM, Zhang LM, Liang YY, Xue W. A star-shaped porphyrin-arginine functionalized poly(L-lysine) copolymer for photo-enhanced drug and gene co-delivery. Biomaterials. 2014;35:4357-67.

[4] Mokhtarzadeh A, Alibakhshi A, Hashemi M, Hejazi M, Hosseini V, de la Guardia M, et al. Biodegradable nano-polymers as delivery vehicles for therapeutic small non-coding ribonucleic acids. Journal of controlled release : official journal of the Controlled Release Society. 2017;245:116-26.
